# A novel glycolysis-related gene signature for predicting prognosis and immunotherapy efficacy in breast cancer

**DOI:** 10.3389/fimmu.2025.1512859

**Published:** 2025-02-19

**Authors:** Rui Huang, Yi Li, Kaige Lin, Luming Zheng, Xiaoru Zhu, Leqiu Huang, Yunhan Ma

**Affiliations:** ^1^ Clinical Laboratory, Jinan Children’s Hospital, Jinan, Shandong, China; ^2^ Clinical Laboratory, Children’s Hospital Affiliated to Shandong University, Jinan, Shandong, China; ^3^ The First Clinical College of Medicine, Wenzhou Medical University, Wenzhou, Zhejiang, China; ^4^ The 960^th^ Hospital of the Chinese People's Liberation Army (PLA) Joint Logistics Support Force, Shandong First Medical University and Shandong Academy of Medical Sciences, Jinan, Shandong, China; ^5^ Department of General Surgery, the 960^th^ Hospital of the Chinese People's Liberation Army (PLA) Joint Logistics Support Force, Jinan, Shandong, China

**Keywords:** bioinformatics, breast cancer, glycolysis, prognostic signature, the cancer genome atlas

## Abstract

**Background:**

Previous studies have shown that glycolysis-related genes (GRGs) are associated with the development of breast cancer (BC), and the prognostic significance of GRGs in BC has been reported. Considering the heterogeneity of BC patients, which makes prognosis difficult to predict, and the fact that glycolysis is regulated by multiple genes, it is important to establish and evaluate new glycolysis-related prediction models in BC.

**Methods:**

In total, 170 GRGs were selected from the GeneCards database. We analyzed data from the Cancer Genome Atlas Breast Invasive Carcinoma (TCGA-BRCA) database as a training set and data from the Gene Expression Omnibus (GEO) database as a validation cohort. Based on the overall survival data and the expression levels of GRGs, Cox regression analyses were applied to develop a glycolysis-related prognostic gene (GRPGs)-based prediction model. Kaplan (KM) survival and ROC analyses were performed to assess the performance of this model. Gene Ontology (GO) and Kyoto Encyclopedia of Genes and Genomes (KEGG) analyses were used to identify the potential biological functions of GRPGs. cBioPortal database was used to explore the tumor mutation burden (TMB). The tumor immune dysfunction and exclusion indicator (TIDE) was used to estimate the patient response to immune checkpoint blockade (ICB). The levels of tumor-infiltrating immune cells (TICs) and stromal cells were quantitatively analyzed based on gene expression profiles.

**Results:**

We constructed a prediction model of 10 GRPGs (ADPGK, HNRNPA1, PGAM1, PIM2, YWHAZ, PTK2, VDAC1, CS, PGK1, and GAPDHS) to predict the survival outcomes of patients with BC. Patients were divided into low- and high-risk groups based on the gene signature. The AUC values of the ROC curves were 0.700 (1-year OS), 0.714 (3-year OS), 0.681 (5-year OS). TMB and TIDE analyses showed that patients in the high-risk group might respond better to ICB. Additionally, by combining the GRPGs signature and clinical characteristics of patients, a novel nomogram was constructed. The AUC values for this combined prediction model were 0.827 (1-year OS), 0.792 (3-year OS), and 0.783 (5-year OS), indicating an outstanding predictive performance.

**Conclusion:**

A new GRPGs based prediction model was built to predict the OS and immunotherapeutic response of patients with BC.

## 1 Introduction

Breast cancer (BC) is the most common malignant tumor in women in the United States, accounting for 31% of all newly diagnosed cancers in women and 15% of cancer-related deaths (1). The incidence of breast cancer has been increasing slowly (1). In China, the incidence and mortality rates of BC in women have shown similar trends (2). With improvements in therapeutics such as adjuvant chemotherapy, targeted treatment, and immunotherapy, BC-related mortality has been reduced. However, the decline in BC mortality has slowed down in recent years (3). Since the heterogeneity of BC led to different therapeutic responses and survival outcomes, there is an urgent need to identify new biomarkers to develop effective risk models to stratify patients using advanced bioinformatics techniques. Several prognostic models have achieved good results in predicting the overall survival (OS) of BC patients (4, 5).

Metabolic rewiring is a well-known hallmark in cancer (6). Metabolites and sufficient energy are necessary for the initiation and proliferation of cancer cells (7). By providing ATP and lactic acid, increased glycolysis can promote cancer progression and drug resistance (8, 9). Under aerobic conditions, non-cancer cells prefer to convert glucose to pyruvate in the first step and thereafter to CO_2_ through mitochondrial oxidation (10). Under hypoxic or anaerobic conditions, cells use glycolysis to convert glucose to lactic acid. On the other hand, cancer cells are inclined to produce large amounts of energy by high glycolytic progress even in the presence of adequate oxygen, which is also a characteristic of BC and is known as the Warburg effect. Many glycolysis-related genes and proteins, including key enzymes in the aerobic glycolytic pathway, have been found to be abnormally expressed in BC and are essential for cancer development. These key enzymes include hexokinase (HK) (11), phosphofructokinase (PFK) (12), pyruvate kinase (PK) (13), and glucose transporters (GLUTs) (14). In addition, activation of some oncogenes [c-myc (15) and HIF-1 (16)] and mutations in tumor suppressors [such as p53 (17)] have also been implicated in the aerobic glycolysis of BC. Several studies have shown that the inhibition of glycolysis can decrease the activity of cancer cells (18, 19). In recent years, a newly identified posttranslational modification (PTM) associated with lactic acid, lactylation, has opened up a new opportunity to investigate the link between glycolysis and epigenetic regulation (20). Thus, a deep understanding of the role of glycolysis in the occurrence and progression of BC may help to predict the prognosis of patients more accurately. Previous studies have investigated glycolysis-related genes and their functions in BC development. A four-glycolysis-gene-based (ALDH2, PRKACB, STMN1, and ZNF292) signature was identified as being related to the recurrence of BC patients (21). A glycolytic expression signature based on another four genes (PGK1, SDHC, PFKL, and NUP43) predicted the survival of BC (22). Another eleven-gene signature related to glycolysis was developed to predict the survival in BC patients (23). Comprehensive research on glycolysis that provides new targets and information is still needed.

Interaction between cancer and stromal cells leads to metabolic competition and symbiosis. Metabolic reprogramming of cancer cells (such as elevated aerobic glycolysis) can shape the metabolism of neighboring cells and vice versa (24). Tumor microenvironment (TME) comprises the extracellular matrix, immune cells (lymphocytes, macrophages, and natural killer cells), stromal cells, and adipocytes, which are important in cancer progression and immunotherapy (25). Immunotherapy has become a key pillar of cancer treatment, the effects of which are associated with the TME (26). Currently, the adverse effects of immunotherapy have prompted an increase in research focused on identifying BC patients who can receive more clinical benefit from immunotherapy using new predictive biomarkers (27).

In our study, we integrated TCGA data and applied univariate and multivariate Cox analyses to identify 10 significant glycolysis-related prognostic genes (GRPGs) in BC. We evaluated the potential of GRPGs as markers associated with survival and prognosis of patients with BC. Notably, GRPGs were successfully employed to construct a nomogram by combining clinical data. The GRPG-based model can predict patient outcomes and immunotherapy efficacy. It showed good performance on different datasets and improved the current BC stratification.

## 2 Materials and methods

### 2.1 Data collection

**Figure 1:**
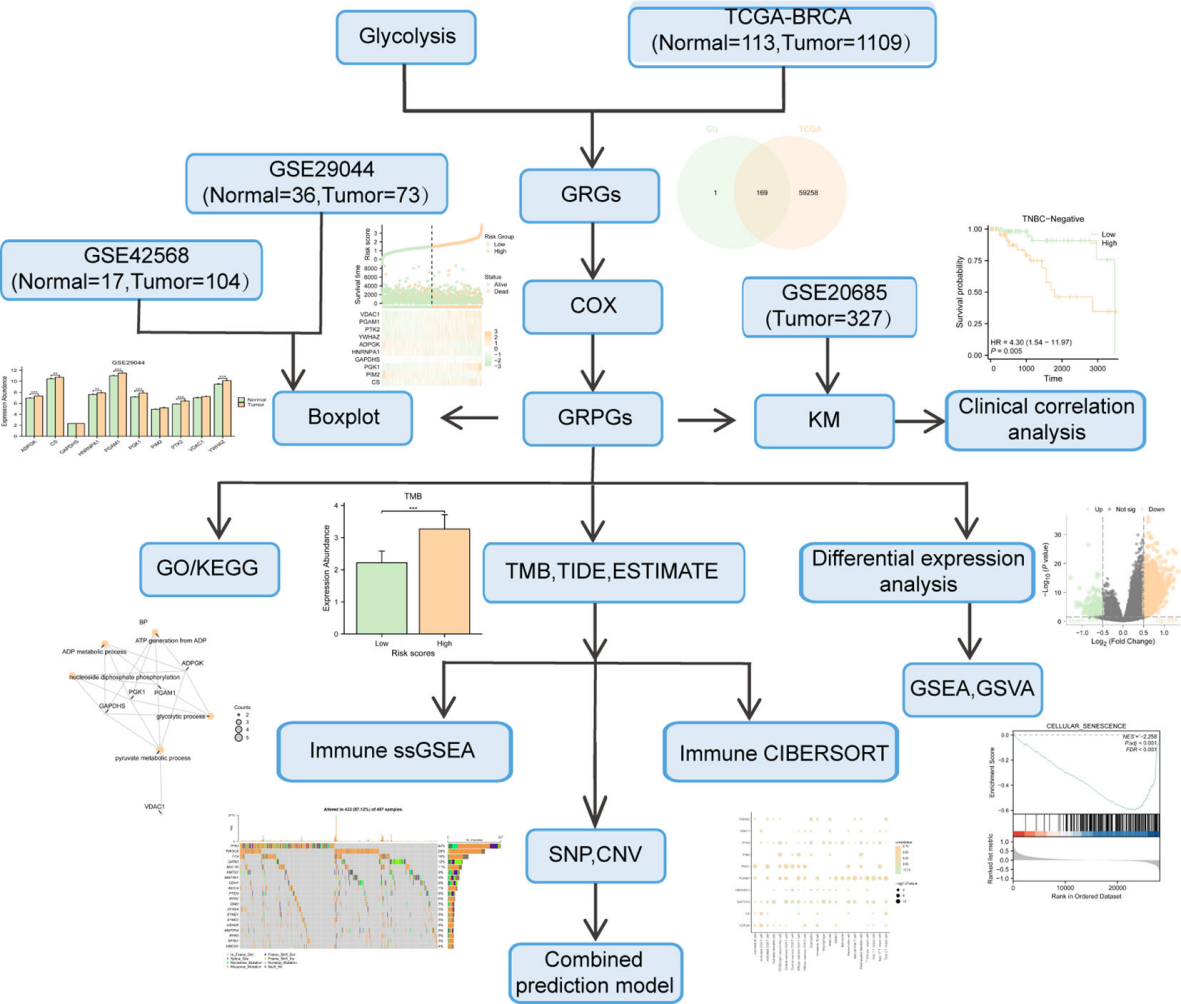
Overall workflow of this study.

The training BC dataset was downloaded from the Cancer Genome Atlas Breast Invasive Carcinoma database (TCGA-BRCA). A total of 1222 count sequencing data of BC samples (1109 tumor samples vs. 113 normal samples) were obtained, which were standardized into Fragments Per Kilobaseper Million (FPKM) format (28). The clinicopathological data corresponding to the samples were downloaded from the UCSC Xena database (http://genome.ucsc.edu) (29), including age, TNM stage, pathologic stage, estrogen receptor (ER) status, progesterone receptor (PR) status, human epidermal growth factor receptor 2 (HER2) status, whether or not triple-negative breast cancer (TNBC) status, and survival outcome (OS, overall survival; DSS, disease-specific survival; PFI, progression-free interval). We also selected the “Masked Somatic Mutation” data from TCGA official website (https://portal.gdc.cancer.gov/) as the somatic mutation data of TCGA-BRCA. In addition, we downloaded the expression profile datasets GSE20685 (30), GSE42568 (31) and GSE29044 (32) of BC patients from the Gene Expression Omnibus (GEO) database. The three datasets were based on the GPL570 platform. GSE20685 contains 327 primary breast cancer samples. GSE42568 and GSE29044 included 121 (104 BC and 17 normal breast) and 109 (73 BC and 36 normal breast) samples, respectively. The GSE20685 dataset was used for verification. Specific information of the TCGA and GEO cohorts is shown in **Supplementary Table S1**. The workflow of this study is illustrated in **Figure 1**.

### 2.2 Construction and evaluation of the 10-GRPGs prediction model

The GeneCards database (33) (https://www.genecards.org/) provides comprehensive information about human genes. We used “glycolysis” as the search keyword to find related genes and only retained “Protein Coding” as well as “Relevance score > 2.00” genes as glycolysis-related genes (GRGs). We obtained 170 GRGs from GeneCards by screening. After removing a gene that was missing from TCGA-BRCA, there were 169 GRGs included in our study (**Supplementary Table S2**).

The expression profile of each GRG in TCGA-BRCA was normalized using a log2 transformation. We combined the expression of 169 GRGs with OS to conduct univariate Cox regression analysis. GRGs significantly associated with OS (*P*<0.05) were identified as glycolysis-related prognostic genes (GRPGs), which were displayed by a forest plot and analyzed by multivariate Cox regression to construct a GRPG-based prediction model. According to the multivariate regression results, we calculated the risk score with coefficients using the following formula: $\text{riskscore }=\;\sum_{\text{i}}\text{Coefficient }\left({\text{hub gene}}_{\text{i}}\right)*\text{mRNA Expression }\left({\text{hub gene}}_{\text{i}}\right)$ . Patients were divided into low- and high-risk groups using the median risk score as the threshold. A risk factor diagram was used to display the risk score distribution, survival status, and expression profile of GRPGs in TCGA-BRCA. Kaplan-Meier (KM) survival analysis was performed to evaluate the difference in OS between the low-risk and high-risk group (34). In order to further assess the performance of this 10-GRPGs prediction model, the time-dependent receiver operating characteristic (ROC) curves were plotted by R package “survivalROC.” The area under the time-dependent ROC curve (AUC) was used to evaluate the predictive efficiency of the model.

### 2.3 Establishment and evaluation of the predictive nomogram

Some clinical parameters affect BC patient prognosis. Cox regression analyses were also performed to demonstrate the independent prognostic value of the 10-GRPGs prediction model and clinical characteristics. We constructed a nomogram by integrating the risk scores of the GRPGs prediction model with clinicopathological variables to evaluate the survival status of patients with BC. At the same time, the prognostic ability of this nomogram was evaluated by ROC, calibration plots and decision curve analysis (DCA). KM analysis was used to prove the prognostic value of this nomogram.

### 2.4 Functional and pathway enrichment analysis

Gene Ontology (GO) analysis (35)is a common method for large-scale functional enrichment research, including biological process (BP), molecular function (MF), and Cellular components (CC). The Kyoto Encyclopedia of Genes and Genomes (KEGG) (36) is a widely used database that stores information about genomes, biological pathways, diseases, and drugs. The R package “clusterProfiler” was used to perform GO and KEGG enrichment analyses to discover the potential biological functions of GRPGs.

### 2.5 Differentially expressed genes, GSEA and GSVA enrichment analysis

Differentially expressed genes (DEGs) in the low-risk and high-risk groups were identified by differential analysis of the expression profile data. Gene set enrichment analysis (GSEA) and gene set variation analysis (GSVA) were used to explore enriched biological pathways.

### 2.6 Immunotherapy efficacy and immune cell infiltration

cBioPortal database (37, 38) (http://cbioportal.org) was used to analyze the tumor mutation burden (TMB) data of BC patients in the TCGA-BRCA. The tumor immune dysfunction and exclusion indicator (TIDE) was used to estimate the patient response to immune checkpoint blockade (ICB). Higher TIDE scores corresponded to greater immune escape and a lower response rate to ICB. To evaluate the relationship between the prediction model and tumor-infiltrating immune cells (TICs), we used ssGSEA (39)to estimate the composition and abundance of TICs. In addition, the levels of TICs and stromal cells in the BC samples were quantitatively analyzed based on their gene expression profiles. The stromal score, immune score, ESTIMATE score, and tumor purity were obtained (40). The correlations between immune cells in different groups were calculated using the Spearman algorithm and visualized using the R package “ggplot2.”

### 2.7 Statistical analysis

All data processing and analyses were performed using the R software (Version 4.1.2). For the comparison of two groups of continuous variables, the statistical significance of normally distributed variables was estimated by independent Student’s t-test, and the Mann-Whitney U test was used (Wilcoxon rank sum test) to analyze the differences among non-normally distributed variables. The “survival” package of R was used for survival analysis, the Kaplan-Meier survival curves were used to show the difference in survival outcomes, and the log-rank test was used to evaluate the significance of the difference in survival time between the two groups. If not specified, *P*<0.05 was considered statistically significant.

## 3 Results

### 3.1 GRPGs and associated prognostic prediction model

**Figure 2:**
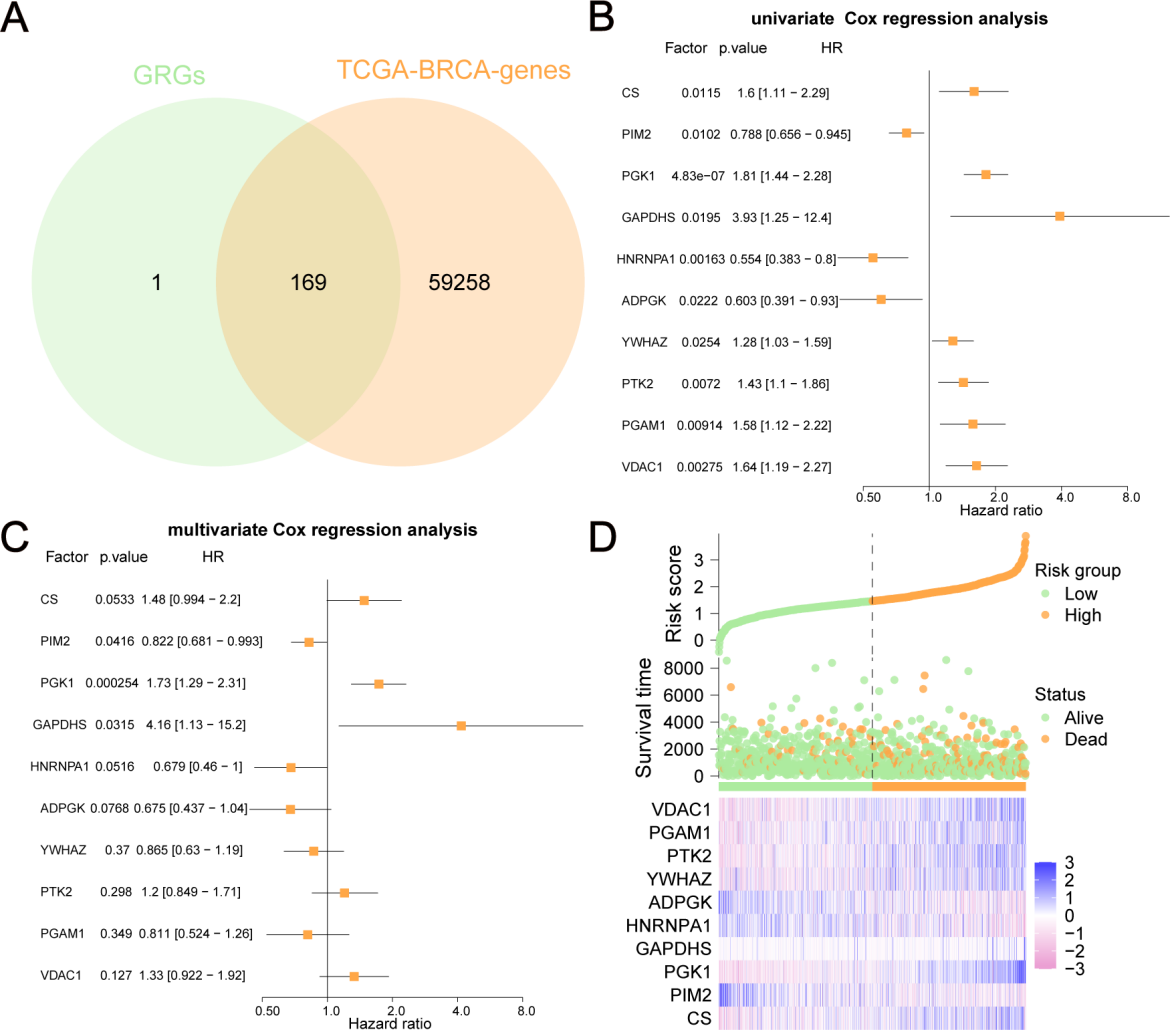
GRPGs selection using univariate and multivariate Cox regression analyses. **(A)** The Venn diagram displayed how 169 GRGs were selected. **(B)** Univariate Cox regression analysis selected 10 GRPGs correlated with OS. **(C)** Multivariate Cox regression analysis result was shown by the forest plot. **(D)** The risk factor diagram showed the risk score distribution, the survival status of BC patients, and the gene expression levels of 10 GRPGs.

By combining GRGs from the GeneCards database with those from TCGA-BRCA, 169 GRGs were selected (**Figure 2A**). We performed univariate and multivariate Cox regression analyses on these GRGs in TCGA-BRCA and found that 10 GRGs (ADPGK, HNRNPA1, PGAM1, PIM2, YWHAZ, PTK2, VDAC1, CS, PGK1, and GAPDHS) were significantly associated with OS (*P*<0.05). These ten GRGs were identified as GRPGs that contributed to the prediction model (**Figures 2B, C**, **Supplementary Table S3**). We calculated the risk scores for BC patients in TCGA-BRCA to evaluate survival risk using the following formula: risk score= (-0.393) × ADPGK + (-0.387) × HNRNPA1 + (-0.209) × PGAM1 + (-0.196) × PIM2 + (-0.145) × YWHAZ + 0.186 × PTK2 + 0.286 × VDAC1 + 0.391 × CS + 0.545 × PGK1 + 1.43 × GAPDHS. With the median risk score as the threshold, patients in TCGA-BRCA were divided into high- and low-risk groups. The risk score distribution, survival status of BC patients, and gene expression levels of the 10 GRPGs are shown in **Figure 2D**.

**Figure 3:**
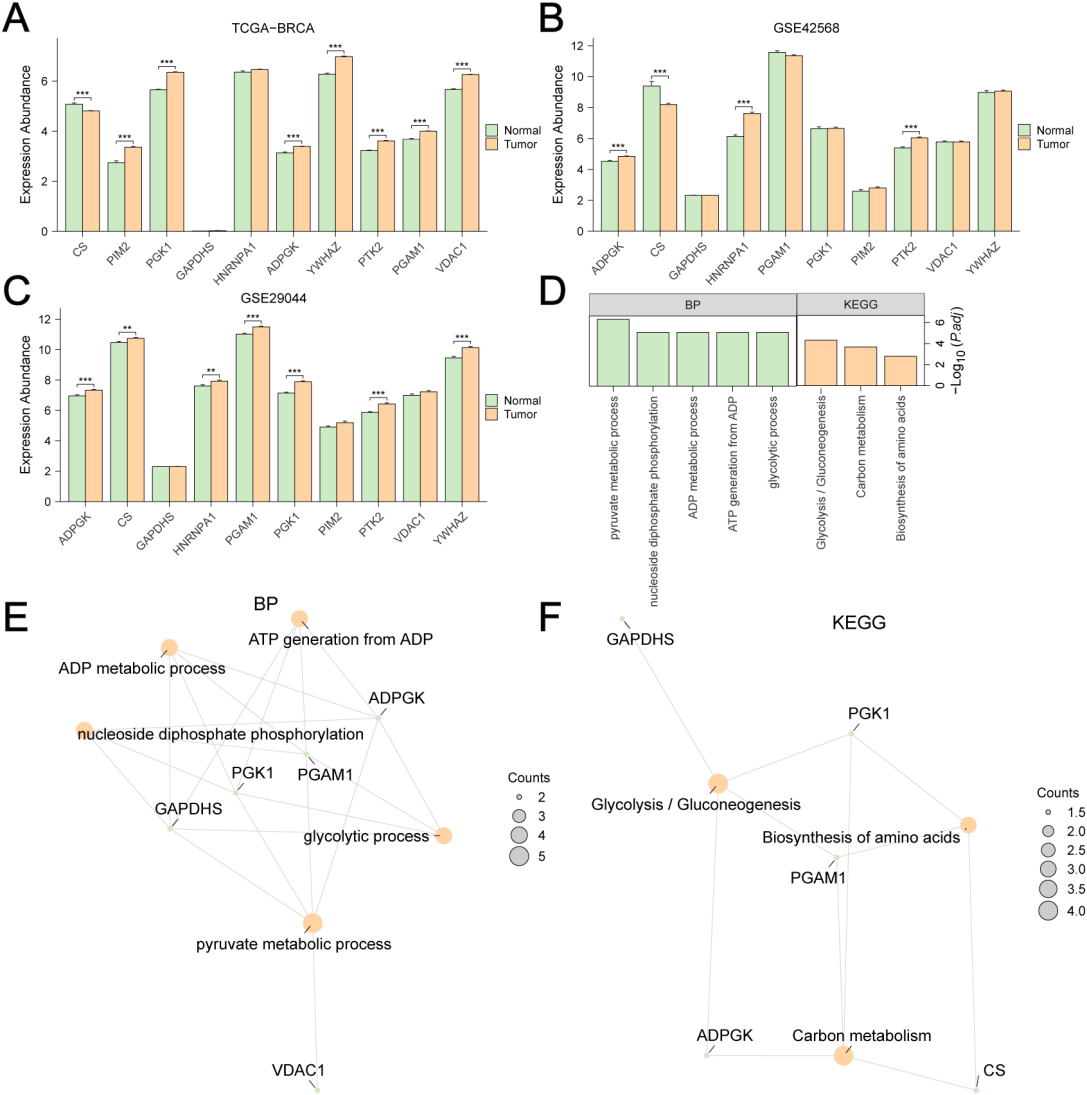
Differential expression and GO/KEGG enrichment analyses of GRPGs. Differential expression analyses of 10 GRPGs between BC tumor and adjacent normal tissues were performed in TCGA-BRCA **(A)**, GSE42568 **(B)**, GSE29044 **(C)**. Biological process (BP) and KEGG enrichment analyses of 10 GRPGs were shown in histogram **(D)** and network diagrams **(E, F)**. **: P value<0.01, ***: P value<0.001.

Differential analyses of the expression of these GRPGs between BC tumors and adjacent normal tissues were also performed in TCGA-BRCA (**Figure 3A**), GSE42568 (**Figure 3B**), and GSE29044 (**Figure 3C**) datasets. Seven GRPGs (PIM2, PGK1, ADPGK, YWHAZ, PTK2, PGAM1, and VDAC1) were significantly upregulated in the TCGA-BRCA tumor tissues. In addition, GSE29044 had more differentially expressed GRPGs between normal and tumor tissues (CS, PGK1, HNRNPA1, ADPGK, YWHAZ, PTK2, and PGAM1) than GSE42568 (CS, HNRNPA1, ADPGK, and PTK2). GO and KEGG enrichment analyses were conducted to predict the biological mechanisms of the GRPGs (**Figure 3D**). GO enrichment analysis showed that the biological process (BP) of GRPGs was mainly involved in pyruvate metabolism, glycolysis, ATP generation from ADP, ADP metabolism, and nucleoside diphosphate phosphorylation (**Figure 3E**, **Supplementary Table S4**). Moreover, KEGG enrichment analysis revealed that GRPGs were also enriched in carbon metabolism, amino acid biosynthesis, and glycolysis/gluconeogenesis pathways (**Figure 3F**, **Supplementary Table S5**). These results indicate that these GRPGs are involved in glycolysis.

### 3.2 The characterization of the two risk groups and survival analysis

**Figure 4:**
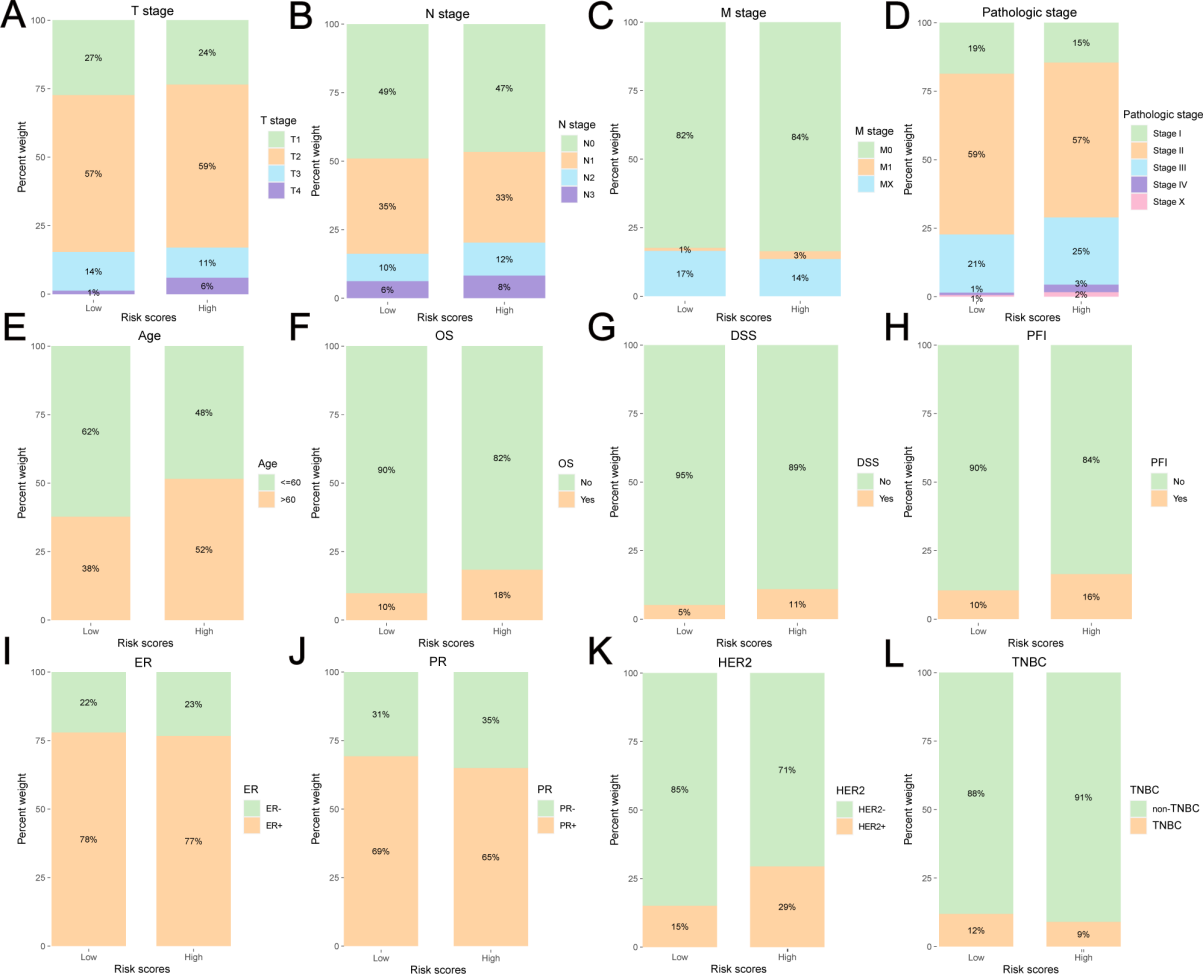
Clinicopathological and survival information of the low-risk and high-risk group for TCGA-BRCA. **(A)** T stage, **(B)** N stage, **(C)** M stage, **(D)** pathologic stage, **(E)** age, **(F)** OS, **(G)** DSS, **(H)** PFI, **(I)**ER status, **(J)** PR status, **(K)** HER2 status, **(L)** TNBC or non-TNBC.

The clinicopathological and survival information of the low-risk and high-risk groups for TCGA-BRCA are shown in **Figures 4A–L** and **Supplementary Table S6**. There were significant differences in T stage (**Figure 4A**), M stage (**Figure 4C**), pathological stage (**Figure 4D**), age (**Figure 4E**), OS (**Figure 4F**), DSS (**Figure 4G**), PFI (**Figure 4H**), and HER2 status (**Figure 4K**) between the two groups (*P*<0.05).

**Figure 5:**
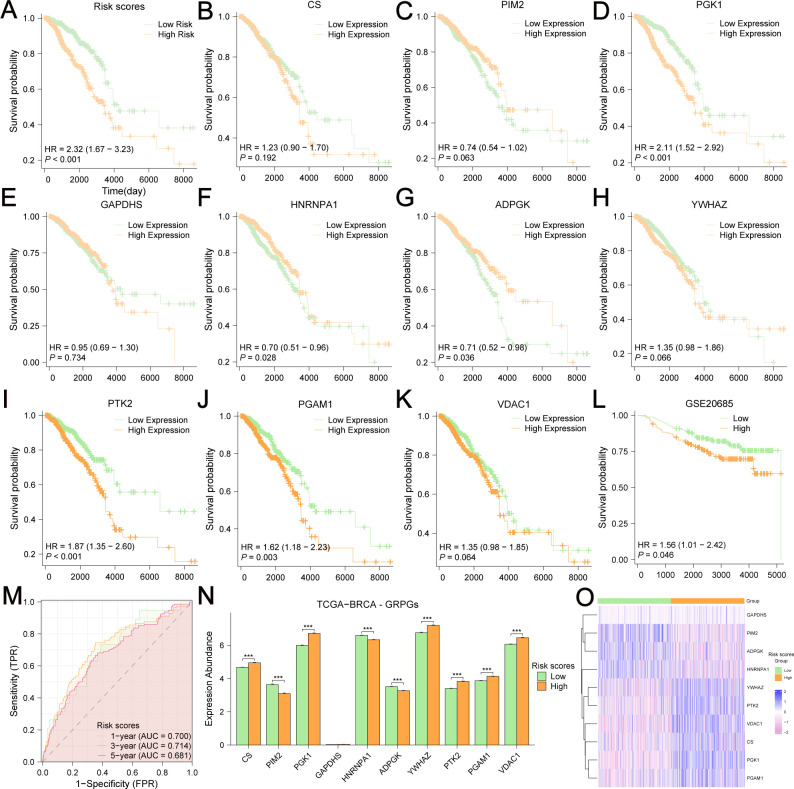
Kaplan-Meier survival analyses in BC patients based on risk stratification and the expression level of each GRPG. The OS difference between low-risk and high-risk group was shown in **(A)**. Kaplan-Meier survival analyses were based on the expression levels of CS **(B)**, PIM2 **(C)**, PGK1 **(D)**, GAPDHS **(E)**, HNRNPA1 **(F)**, ADPGK **(G)**, YWHAZ **(H)**, PTK2 **(I)**, PGAM1 **(J)**, VDAC1 **(K)** in TCGA-BRCA. **(L)** The OS difference between low-risk and high-risk group in GSE20685 was displayed by the KM curves. **(M)** AUC values were calculated in ROC analysis for risk scores predicting the OS from TCGA-BRCA. **(N)** Differential expression analyses of 10 GRPGs between low-risk group and high-risk group were performed in TCGA-BRCA. **(O)** The expression patterns of 10 GRPGs were shown in the heatmap.

The Kaplan-Meier survival curve showed that the high-risk group had a poorer outcome than the low-risk group (**Figure 5A**). We also assessed the relationship between the expression level of each GRPG and OS in patients (**Figures 5B–K**). We found that the survival differences of the low-expression and high-expression groups of five GRPGs (PGK1, ADPGK, PTK2, PGAM1, and HNRNPA1) were significant (*P*<0.05). To evaluate the robustness of this 10-GRPGs signature, we used GSE20685 as a validation cohort to assess its performance. Similar to the results of TCGA-BRCA, BC patients in the high-risk group had a worse prognosis (**Figure 5L**). The AUC values of the ROC curves for TCGA-BRCA were 0.700 (1-year OS), 0.714 (3-year OS), and 0.681 (5-year OS) (**Figure 5M**), indicating the predictive ability of this prediction model. In addition, differential analyses of the expression of 10 GRPGs between the low- and high-risk groups were conducted using TCGA-BRCA. Significant differences were observed in almost all GRPGs between these two groups, except for GAPDHS (**Figure 5N**). The expression patterns of the 10 GRPGs in the low- and high-risk groups are shown in the heatmap (**Figure 5O**).

**Figure 6:**
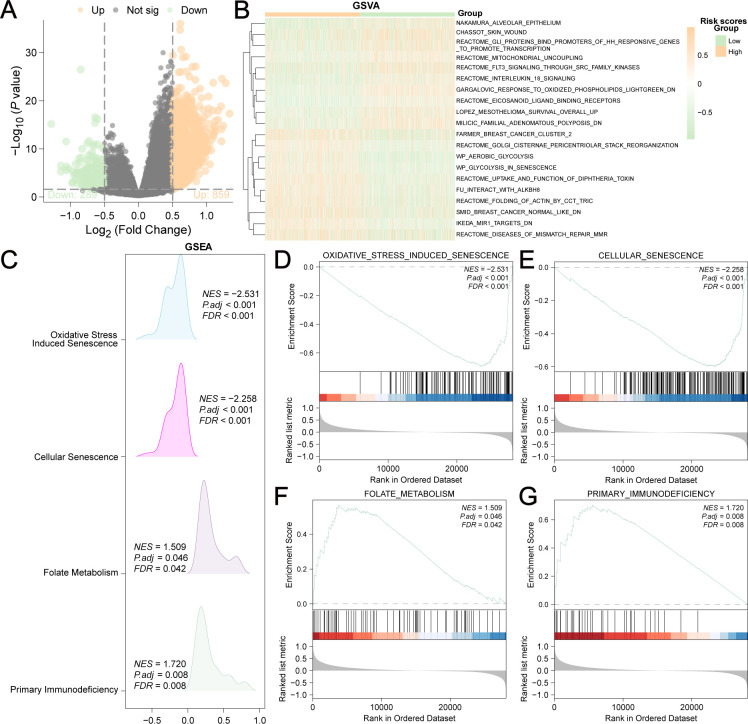
Differentially expressed genes (DEGs) of low-risk group versus high-risk group. **(A)** 1148 DEGs were shown in the Volcano plot (|logFC| > 0.5 and adjusted P<0.05). **(B)** 20 enriched biological functions obtained by GSVA analysis were shown in the heatmap. **(C)** Mountain plot showed the four main biological features of DEGs achieved by GSEA enrichment analysis. DEGs were significantly enriched in oxidative stress induced senescence **(D)**, cellular senescence **(E)**, folate metabolism **(F)** and primary immunodeficiency **(G)**.

The differentially expressed genes (DEGs) of the low-risk group versus the high-risk group were analyzed, and 1148 DEGs were identified (|logFC|>0.5 and adjusted *P*<0.05) (**Figure 6A**). We used to explore the biological functions of these DEGs. The related functions at the top of the list are shown in **Figure 6B** and **Supplementary Table S7**. In addition, GSEA (**Figure 6C**, **Supplementary Table S8**) revealed that these DEGs were significantly enriched in four biological pathways: oxidative stress-induced senescence (**Figure 6D**), cellular senescence (**Figure 6E**), folate metabolism (**Figure 6F**), and primary immunodeficiency (**Figure 6G**).

**Figure 7:**
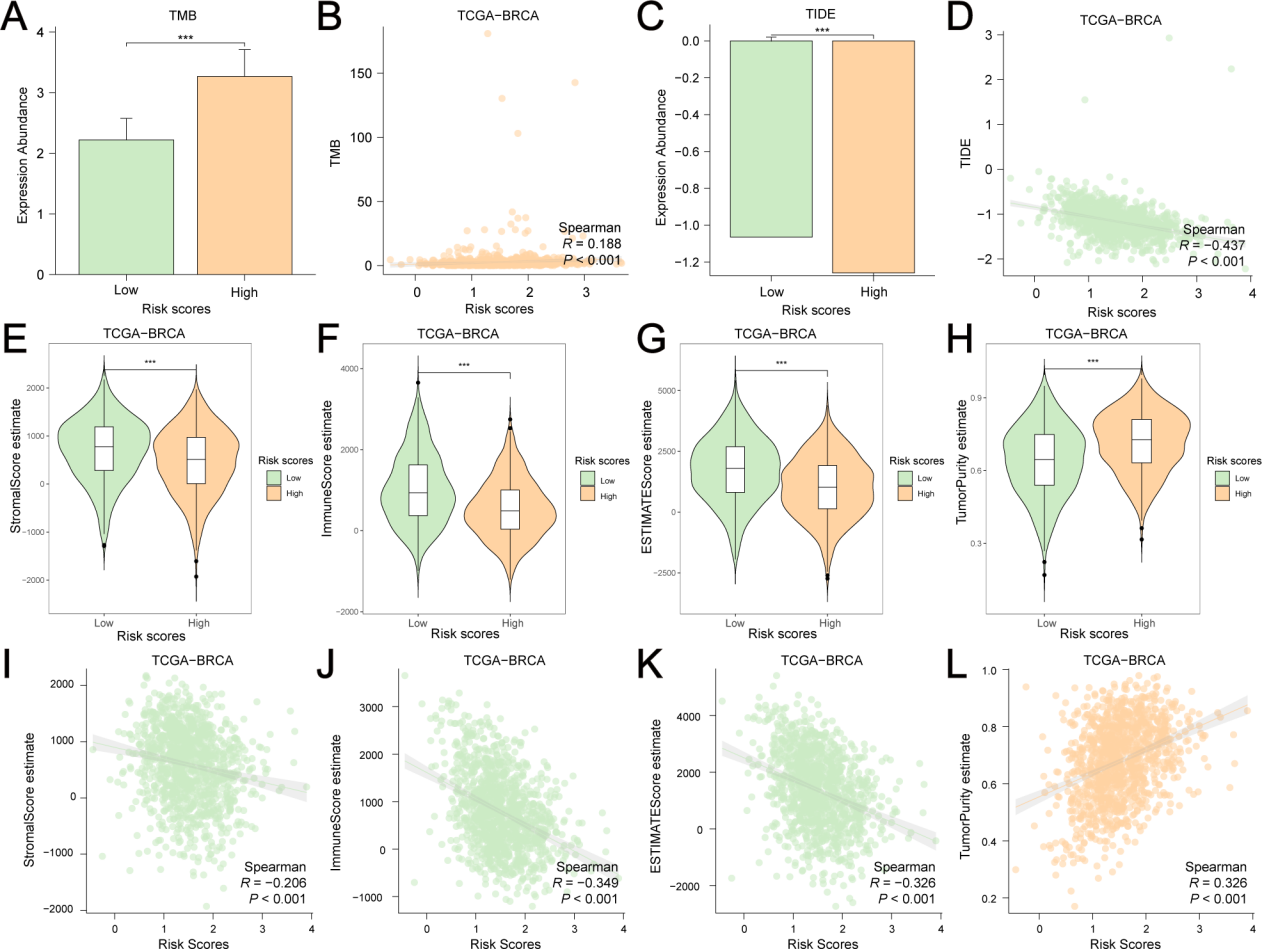
Estimation of TMB, TIDE, stromal score, immune score, ESTIMATE score and tumor purity score in low-risk and high-risk group. **(A)** The histogram showed the differential TMB scores between the low-risk and high-risk group. **(B)** A positive correlation between risk scores and TMB scores was found by Spearman correlation test. **(C)** The histogram showed the differential TIDE scores between the low-risk and high-risk group. **(D)** A negative correlation between risk scores and TMB scores was found by Spearman correlation test. Violin plots showed the differential stromal score **(E)**, immune score **(F)**, ESTIMATE score **(G)** and tumor purity score **(H)** between the high-risk and low-risk groups. Risk scores were negatively correlated with stromal score **(I)**, immune score **(J)** and ESTIMATE score **(K)** but were positively correlated with tumor purity score **(L)**. ***: P value<0.001.

**Figure 8:**
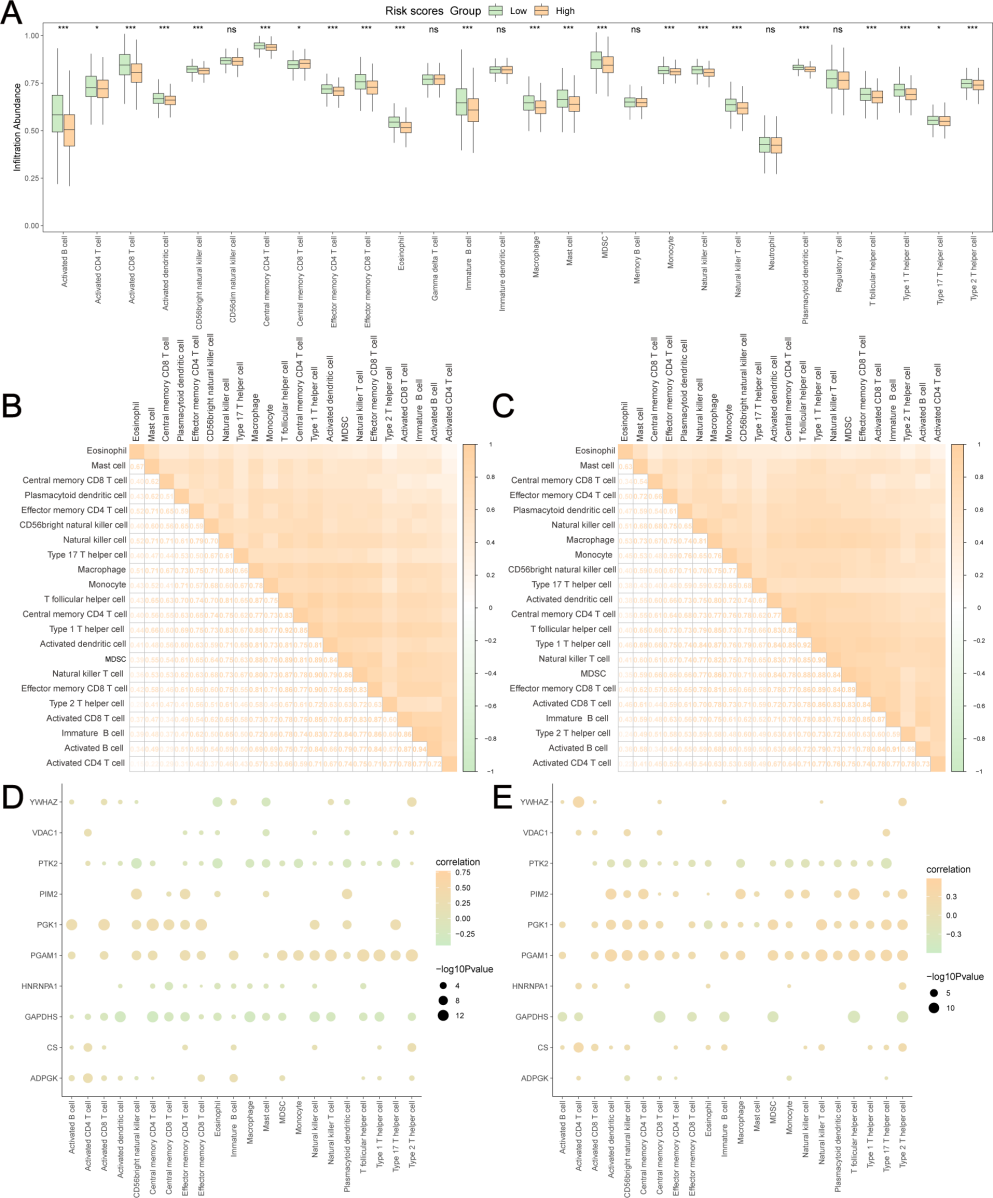
Estimation of TICs in low-risk and high-risk group. **(A)** The differences of 28 TICs between low-risk and high-risk group were evaluated by ssGSEA algorithm. The correlation analyses of TICs were conducted in low-risk group **(B)** and high-risk group **(C)**. The dot plots showed the correlation between the abundance of TICs and the expression levels of GRPGs in low-risk group **(D)** and high-risk group **(E)**. *: P value<0.05, ***: P value<0.001, ns: P values≥0.05.

GRPGs have a potential role in predicting response to immunotherapy in BC patients. The result showed that high-risk group had a higher TMB score than the low-risk group, suggesting that the high-risk group had a better response to immunotherapy (**Figure 7A**). A positive correlation between the risk scores and TMB scores was found for TCGA-BRCA (**Figure 7B**). Similarly, the high-risk group had a lower TIDE score, indicating that the high-risk group had a higher response rate to ICB (**Figure 7C**). The risk scores were negatively correlated with the TIDE scores (**Figure 7D**). To evaluate the relative abundance of cancer cells, immune cells, and stromal cells, we used the “estimate” package in R to calculate stromal scores, immune scores, ESTIMATE scores, and tumor purity scores. The high-risk group showed significantly lower stromal and immune scores and ESTIMATE scores, but higher tumor purity scores than the low-risk group (**Figures 7E–H**). In addition, risk scores were negatively correlated with stromal, immune, and ESTIMATE scores, but positively correlated with tumor purity scores (**Figures 7I–L**). The above results indicated that BC in the low-risk group had more immune and stromal cell abundance. Next, we estimated the quantified abundance of many immune cell types using ssGSEA to determine the TICs differences in the low-risk and high-risk groups. According to ssGSEA, the infiltration of 21 immune cell types, including activated B cells, activated CD4^+^ T cells, activated CD8^+^ T cells, activated dendritic cells, and macrophages, was significantly higher in the low-risk group than in the high-risk group. Only the number of central memory CD8^+^ T cells was lower in the low-risk group (**Figure 8A**). Correlation analyses of TICs showed that the abundance of TICs was mostly positively correlated in the low-risk group (**Figure 8B**) or in the high-risk group (**Figure 8C**). The dot plots of the correlation between TICs and GRPGs revealed that the expression levels of PIM2, PGK1, PGAM1, and CS were positively correlated with the abundance of TICs, whereas the expression levels of PTK2 and GAPDHS were negatively correlated with the abundance of TICs (**Figures 8D, E**). Taken together, these results indicate the effectiveness of risk score in BC immunotherapy and immune cell infiltration prediction.

**Figure 9:**
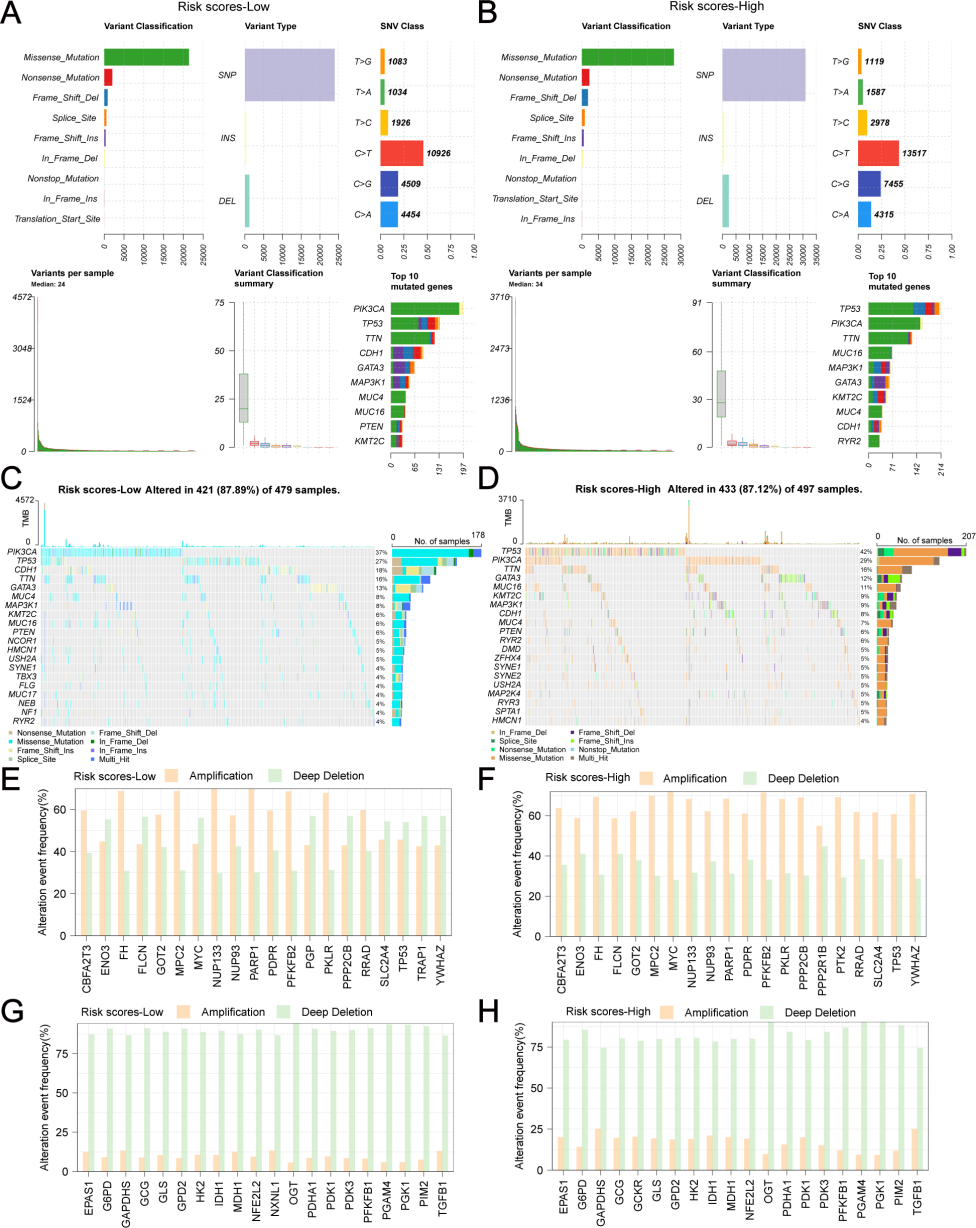
Genetic alterations of GRPGs in low-risk and high-risk group. The combined graph displayed the variant classification, variant types, single nucleotide variations (SNV) class, the number of variants and top 10 mutated genes in low-risk group **(A)** and high-risk group **(B)**. The waterfall plots showed the genetic alterations of GRPGs sorted by mutation rate in low-risk group **(C)** and high-risk group **(D)**. The histograms showed the top 20 gene amplifications in low-risk group **(E)** and high-risk group **(F)**. The histograms showed the top 20 gene deletions in low-risk group **(G)** and high-risk group **(H)**.

We analyzed the genetic alterations of GRPGs in both groups and found that the main types of alterations included missense mutations, nonsense mutations, frameshift dels, frameshift ins, splice sites, in-frame dels, and in-frame ins. TP53, PIK3CA, TTN, CDH1, GATA3, MAP3K1, MUC4, MUC16, and KMT2C were the GRPGs with the highest mutational abundance in both groups (**Figures 9A, B**, sorted by the total number of mutation sites). Waterfall plots showed that five GRPGs (PIK3CA, TP53, CDH1, TTN, GATA3) were commonly mutated (>10%) in the low-risk group samples (**Figure 9C**), whereas five GRPGs (TP53, PIK3CA, TTN, GATA3, and MUC16) were commonly altered (>10%) in the high-risk group samples (**Figure 9D**). We then analyzed the copy number variations (CNV) of these GRPGs. **Figures 9E, F** show the top 20 gene amplifications in the low-risk and high-risk groups, respectively. NUP133 and PARP1 had the highest amplification frequency in the low-risk group, whereas MYC and PFKFB2 were amplified in the high-risk group. **Figures 9G, H** show the top 20 gene deletions in the low-risk and high-risk groups, respectively. OGT and PGAM4 had the highest deletion frequencies in both groups.

### 3.3 Data stratification and subgroup analyses

**Figure 10:**
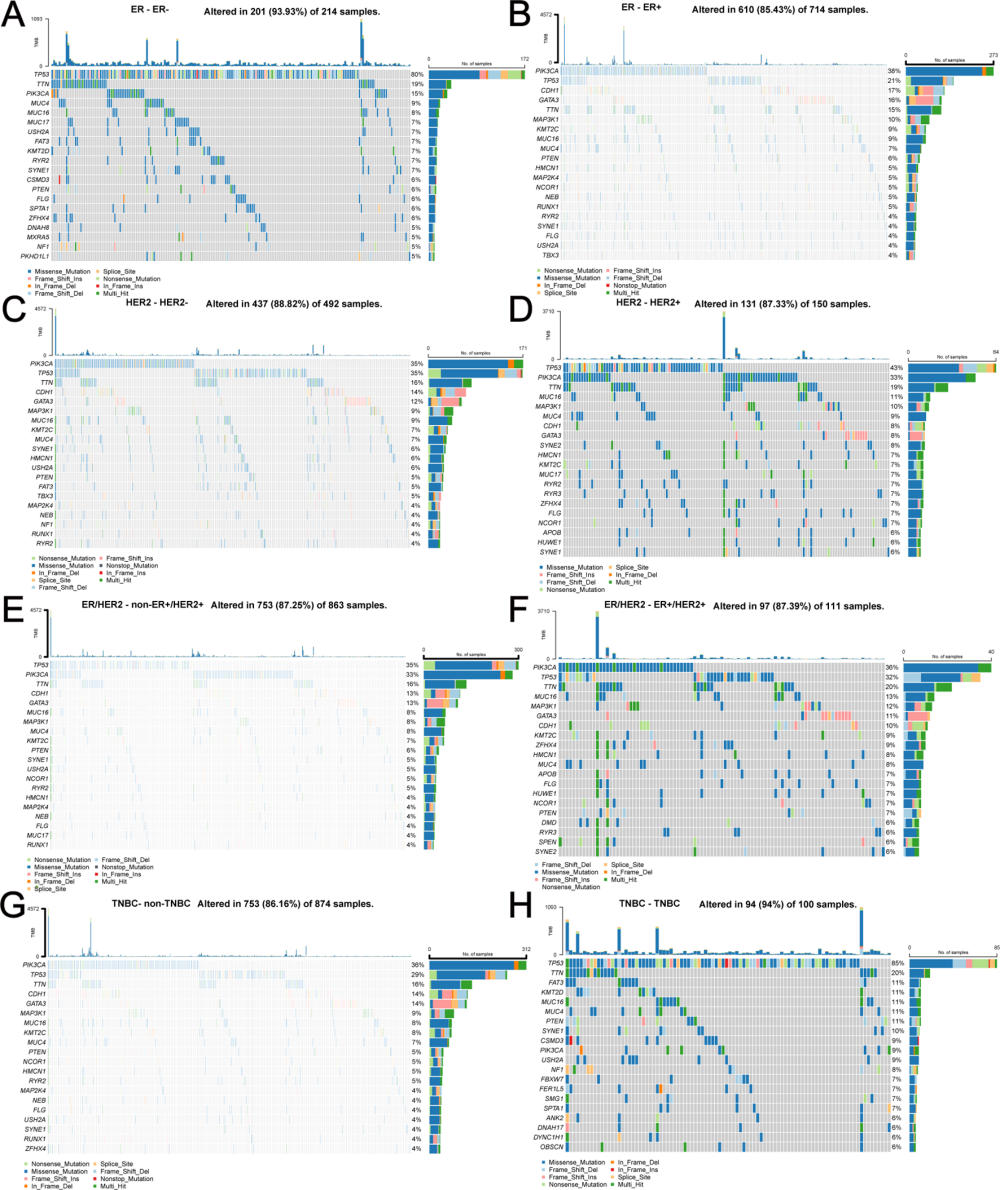
Specific genetic alterations and corresponding number of samples depending on different molecular types of BC. **(A)** ER-, **(B)** ER+, **(C)** HER2-, **(D)** HER2+, **(E)** non-ER+HER2+, **(F)** ER+HER2+, **(G)** non-TNBC, **(H)** TNBC.

Depending on the molecular type of BC (ER+/-, HER2+/-, ER+HER2+/non-ER+HER2+, TNBC/non-TNBC), specific genetic alterations and the corresponding number of samples are shown in **Figures 10A–H**. Regardless of the molecular type of BC, the number of samples harboring TP53 and PIK3CA mutations is always at the forefront.

**Figure 11:**
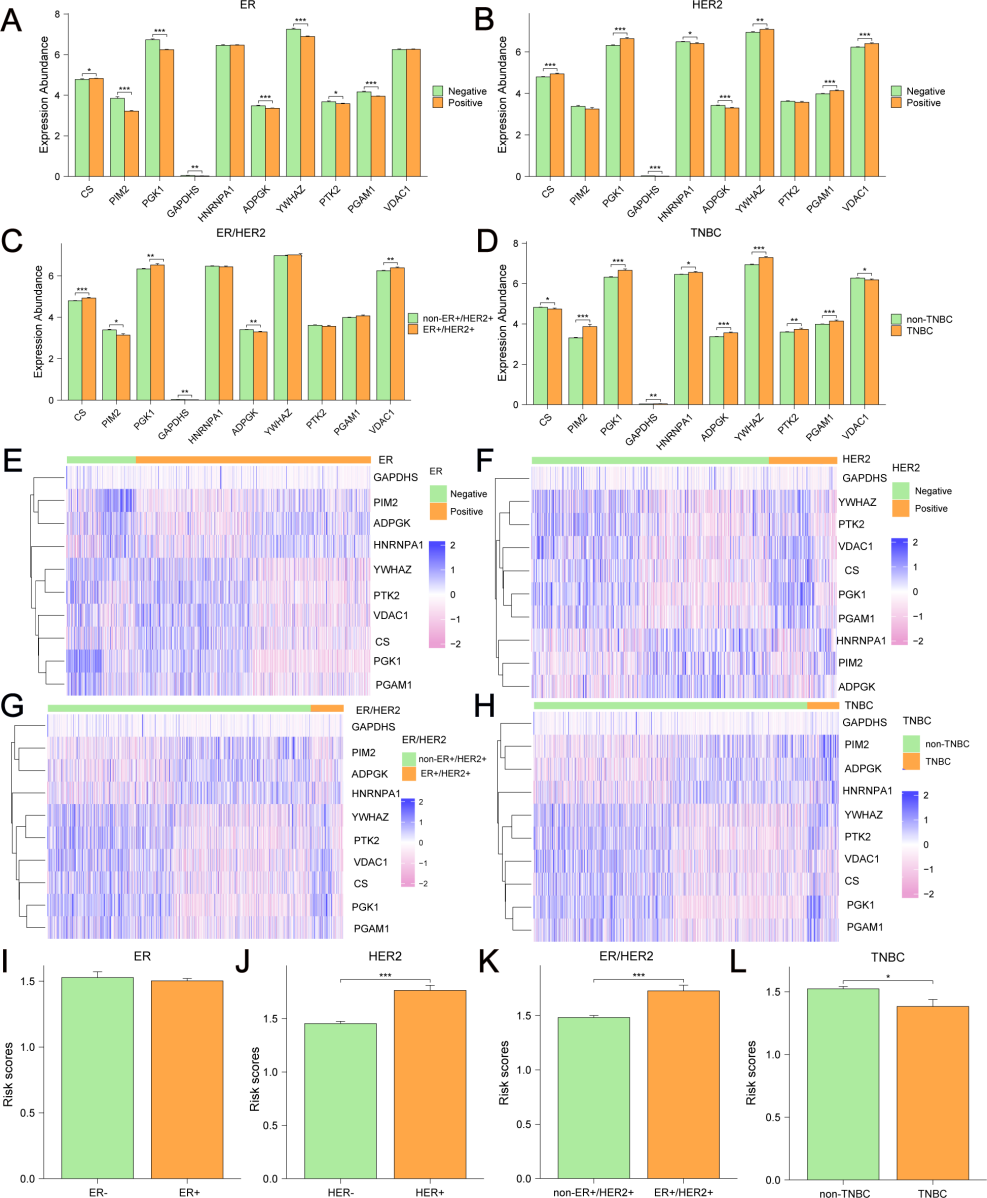
Differential expression of 10 GRPGs and risk scores in different BC subgroups. Differential analyses of the expression of 10 GRPGs was conducted in ER+/- BC **(A)**, HER2+/- BC **(B)**, ER+HER2+/non-ER+HER2+ BC **(C)** and TNBC/non-TNBC **(D)**. The heatmaps showed the expression patterns of 10 GRPGs in ER+/- BC **(E)**, HER2+/- BC **(F)**, ER+HER2+/non-ER+HER2+ BC **(G)** and TNBC/non-TNBC **(H)**. The histograms showed risk scores in ER+/- BC **(I)**, HER2+/- BC **(J)**, ER+HER2+/non-ER+HER2+ BC **(K)** and TNBC/non-TNBC **(L)**. *: P value<0.05, **: P value<0.01, ***: P value<0.001.

Differential analyses of the expression of 10 GRPGs in different molecular types of BC were conducted using TCGA-BRCA. Significant differences existed in 8, 8, 6, and 10 GRPGs between the ER+ and ER- subgroups (**Figure 11A**), HER2+ and HER2- subgroups (**Figure 11B**), ER+HER2+ and non-ER+HER2+ subgroups (**Figure 11C**), and TNBC and non-TNBC subgroups (**Figure 11D**), respectively. The expression patterns of 10 GRPGs in BC of different molecular types are shown in heatmaps (**Figures 11E–H**). In addition, HER2+ BC, ER+HER2+BC, and non-TNBC BC patients showed significantly higher risk scores than HER2- BC, non-ER+HER2+BC, and TNBC BC patients, respectively (**Figures 11I–L**).

**Figure 12:**
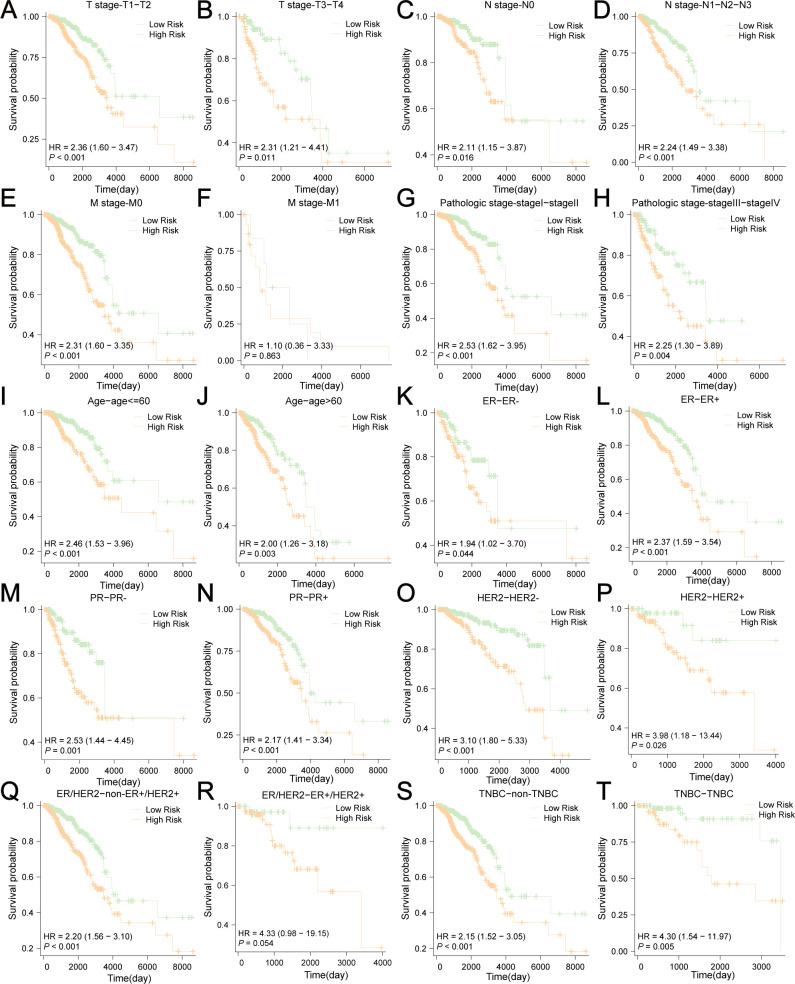
Survival subgroup analyses based on the clinicopathological variables. **(A)** T1-T2 stage, **(B)** T3-4 stage, **(C)** N0 stage, **(D)** N (+) stage (N1-N3), **(E)** M0 stage, **(F)** M1 stage, **(G)** pathologic stage I-II, **(H)** pathologic stage III-IV, **(I)** age ≤ 60, **(J)** age>60, **(K)** ER-, **(L)** ER+, **(M)** PR-, **(N)** PR+, **(O)** HER2-, **(P)** HER2+, **(Q)** non-ER+HER2+, **(R)** ER+HER2+, **(S)** non-TNBC, **(T)** TNBC.

Next, we conducted survival subgroup analyses based on clinicopathological variables. Patients from TCGA-BRCA were classified into different subgroups: T1-T2 stage vs. T3-4 stage; N0 stage vs. N (+) stage (N1-N3); M0 vs. M1 stage, pathologic stage I-II vs. pathologic stage III-IV, age ≤ 60 vs. age>60, ER- vs. ER+, PR- vs. PR+, HER2- vs. HER2+, non-ER+HER2+ vs. ER+HER2+, and non-TNBC vs. TNBC. The KM curves indicated that risk scores showed excellent ability to predict prognoses in BC patients stratified by T/N stage, pathologic stage, age, ER status, PR status, HER2 status, and non-TNBC/TNBC status (**Figures 12A–T**). Nevertheless, no significant survival difference was observed between the low-risk and high-risk groups in M1 (*P*=0.863) and ER+HER2+ patients (*P*=0.054). These results indicate that the risk scores had good predictive value in different clinical subgroups.

### 3.4 Establishment of a nomogram for clinical application

**Figure 13:**
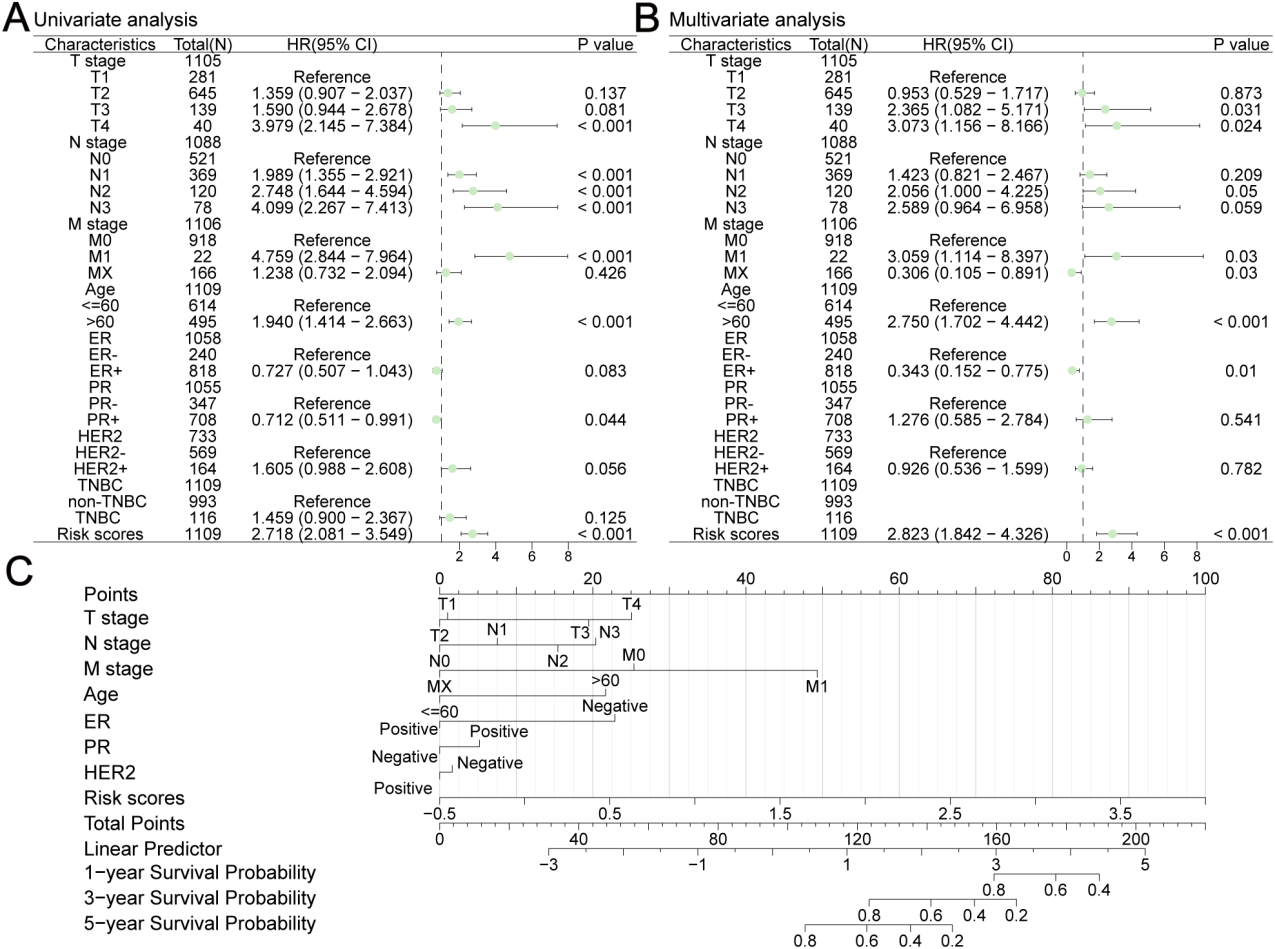
Construction a nomogram in TCGA-BRCA. Univariate **(A)** and multivariate **(B)** Cox analyses were performed to analyze several clinicopathological data. **(C)** The nomogram consisted of TNM stage, age, ER status, PR status, HER2 status and risk scores to predict the probability of 1-year, 3-year and 5-year OS.

We integrated risk scores with other clinical risk factors in TCGA-BRCA to further evaluate the independent prognostic value of our prediction model. Univariate and multivariate Cox analyses were performed to analyze the clinicopathological data (**Figures 13A, B**, **Table 1**). TNM stage, age, ER status, PR status, HER2 status, and risk scores were effective predictors of OS in the univariate Cox analyses (*P* < 0.1). We combined these clinical factors and risk scores to evaluate survival risk by calculating prognostic combined risk scores: Combined risk scores = stageT2×-0.04816+stageT3×-0.86080+stageT4×1.12257+stageN1×0.35266+stageN2×0.72070+stageN3×0.95139+stageM1×1.11801+stageMX×-1.18466+Age>60×1.01152+ERPositive×-1.06914+PRPositive×0.24354+HER2Positive×-0.07732+ risk scores×1.03767-1.57445. The constant value (-1.57445) can effectively adjust baseline risk, so that the combined risk score output by this model can reflect the true prognosis of patients. The introduction of this constant value takes into account the potential influencing factors that are not included in this model, making the combined risk scores of different patients comparable and providing some support for the interpretability of this model.

**Table 1:** COX analyses of several clinicopathological data.

Characteristics	Total (N)	Univariate analysis		Multivariate analysis	
		Hazard ratio (95% CI)	P value	Hazard ratio (95% CI)	P value
T stage	1,105				
T1	281	Reference		Reference	
T2	645	1.359 (0.907 - 2.037)	0.137	0.953 (0.529 - 1.717)	0.873
T3	139	1.590 (0.944 - 2.678)	0.081	2.365 (1.082 - 5.171)	**0.031**
T4	40	3.979 (2.145 - 7.384)	**< 0.001**	3.073 (1.156 - 8.166)	**0.024**
N stage	1,088				
N0	521	Reference		Reference	
N1	369	1.989 (1.355 - 2.921)	**< 0.001**	1.423 (0.821 - 2.467)	0.209
N2	120	2.748 (1.644 - 4.594)	**< 0.001**	2.056 (1.000 - 4.225)	0.050
N3	78	4.099 (2.267 - 7.413)	**< 0.001**	2.589 (0.964 - 6.958)	0.059
M stage	1,106				
M0	918	Reference		Reference	
M1	22	4.759 (2.844 - 7.964)	**< 0.001**	3.059 (1.114 - 8.397)	**0.030**
MX	166	1.238 (0.732 - 2.094)	0.426	0.306 (0.105 - 0.891)	**0.030**
Age	1,109				
<=60	614	Reference		Reference	
>60	495	1.940 (1.414 - 2.663)	**< 0.001**	2.750 (1.702 - 4.442)	**< 0.001**
ER	1,058				
ER-	240	Reference		Reference	
ER+	818	0.727 (0.507 - 1.043)	0.083	0.343 (0.152 - 0.775)	**0.010**
PR	1,055				
PR-	347	Reference		Reference	
PR+	708	0.712 (0.511 - 0.991)	**0.044**	1.276 (0.585 - 2.784)	0.541
HER2	733				
HER2-	569	Reference		Reference	
HER2+	164	1.605 (0.988 - 2.608)	0.056	0.926 (0.536 - 1.599)	0.782
TNBC	1,109				
non-TNBC	993	Reference			
TNBC	116	1.459 (0.900 - 2.367)	0.125		
Risk scores	1,109	2.718 (2.081 - 3.549)	**< 0.001**	2.823 (1.842 - 4.326)	**< 0.001**

Bold values: P value<0.05.

**Figure 14:**
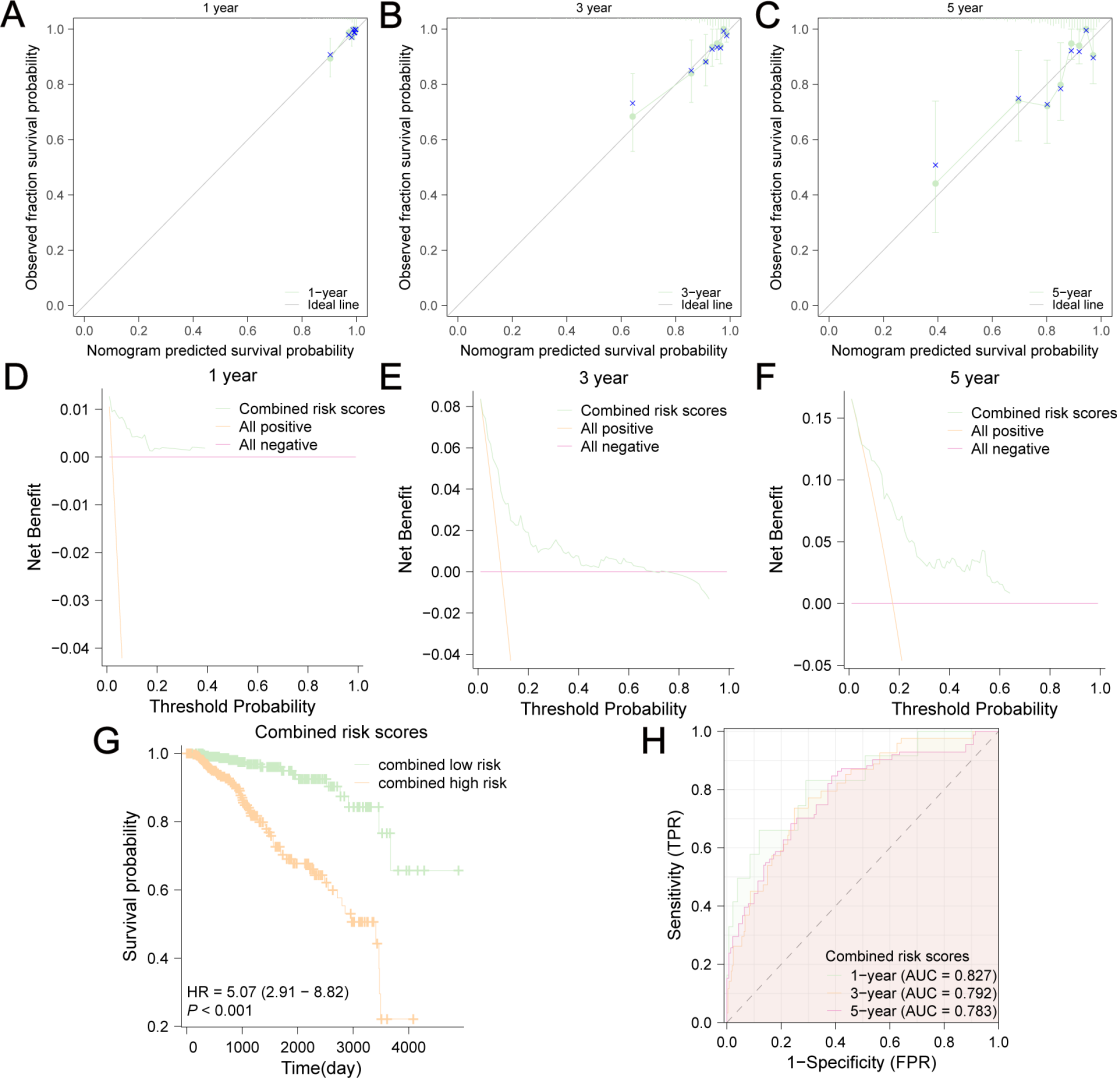
Evaluation of this nomogram. Calibration curves of 1-year **(A)**, 3-year **(B)** and 5-year **(C)** OS predicted by the nomogram showed the relationship between predicted survival probability and observed fraction survival probability. DCA curves of 1-year **(D)**, 3-year **(E)** and 5-year **(F)** OS prediction showed the clinical predictive effects of this combined prediction model. The OS difference between combined low-risk and high-risk group was shown in **(G)**. **(H)** AUC values were calculated in ROC analysis for combined risk scores predicting the OS from TCGA-BRCA.

To facilitate clinical application, we built a visualized nomogram to predict the 1-year, 3-year and 5-year OS of patients with BC (**Figure 13C**). These clinical factors were included in the nomogram as parameters. The calibration curves (**Figures 14A–C**) indicated that the predicted curves were in good agreement with the ideal curves. DCA curves showed that this combined prediction model had good clinical predictive effects (**Figures 14D–F**). Using the median combined risk score as the threshold, patients were further divided into a combined high-risk group and a combined low-risk group. KM curves revealed that BC patients with a combined high-risk score had poorer outcomes than those in the combined low-risk group (**Figure 14G**). The AUC values of the ROC curves for this combined prediction model were 0.827 (1-year OS), 0.792 (3-year OS), and 0.783 (5-year OS) (**Figure 14H**), confirming the reliability of our study.

## 4 Discussion

Heterogeneity, a characteristic of breast cancer (BC) with diverse phenotypes and morphologies, makes it difficult to predict the prognosis of patients (41). Altered glucose metabolism exists in all BC types, which plays an important role in driving cancer progression and therapy resistance. Reprogramming of BC glucose metabolism is characterized by hyperactivity of glycolysis and accumulation of lactate. An increase in aerobic glycolysis, known as the Warburg effect, which is induced by the upregulation of key glycolytic enzymes and glucose transporters, can provide BC cells with ATP and an acidic microenvironment (42). An increasing number of genetic signatures has been explored to improve the ability to predict the prognosis of BC patients. Although the prognostic significance of GRGs in BC has been reported (21, 22), considering that glycolysis is a multi-step enzymatic reaction that is regulated by multiple genes, a new GRPG-based signature for predicting BC patient prognosis is needed. Furthermore, the emergence of a novel epigenetic modification (43), lactylation, has made glycolysis and lactate research focus again. It is necessary to expand our knowledge of GRPGs to explore the underlying mechanisms. Thus, it is important to establish and assess glycolysis-related prediction models for BC.

In our study, we built a GRPG-based model to predict the survival outcomes of BC patients and provided risk stratification. We searched the GeneCards database for genes related to glycolysis, which were used in subsequent analyses. The OS data from TCGA-BRCA were used to perform univariate and multivariate Cox regression analyses to identify GRPGs, which included 10 hub genes. ADPGK, HNRNPA1, PGAM1, PIM2, and YWHAZ were positively associated with survival, while the expression levels of PTK2, VDAC1, CS, PGK1and GAPDHS were negatively associated with survival. Based on these GRPGs, we verified this prediction model using TCGA-BRCA and GEO datasets. KM survival analyses revealed different prognoses between the high-risk and low-risk groups, demonstrating the favorable survival predictive ability of this model. The time-dependent ROC curves also confirmed the good predictive performance of these 10 GRPGs. We also found that patients in the high-risk group had different clinical parameters than those in the low-risk group, including age, T stage, M stage, pathologic stage, HER2 status, OS, DSS, and PFI. Moreover, the risk score originating from these GRPGs could further stratify clinically defined patients into low- and high-risk groups with different OS. Through subgroup analyses, we found that this model could accurately predict survival in subgroups stratified according to T/N stage, pathologic stage, age, ER status, PR status, HER2 status, and non-TNBC/TNBC status, but it might not be applicable to M1 patients and ER+HER2+ patients. In addition, the risk score can be regarded as an independent prognostic factor. We integrated the data and clinical characteristics to build a novel nomogram that utilized the values of age, TNM stage, ER status, PR status, HER2 status, and risk scores. This nomogram exhibited superior power and accuracy of estimation with a higher AUC, suggesting that the combination of risk score with clinical risk factors is more effective for OS prediction. These results demonstrated that the GRPG-based prediction model in our study had good prognostic significance. In a previous glycolysis-related gene signature, the gene expression profiles and clinical data of breast cancer patients were obtained from the GEO database. A four-gene based signature (ALDH2, PRKACB, STMN1 and ZNF292) was developed to separate patients into high-risk and low-risk groups. High expression level of the PRKACB protein was associated with favorable prognosis, while high ZNF292 and STMN1 protein expression levels indicated poor prognosis (21). In a glycolysis-related 4-mRNA signature study for predicting the survival of patients with breast cancer (22), the AUC values were 0.74 (training cohort), 0.806 (testing cohort) and 0.769 (entire cohort). The AUC of the nomogram based on clinical data and 4-mRNA signature risk score at 3-year and 5-year was 0.808 and 0.755, respectively. The prediction performance of the 4-mRNA signature study was comparable to ours. Another 11-gene signature related to glycolysis for predicting survival in patients with BC was developed. The authors analyzed the data of a training set from TCGA database and four validation cohorts from the GEO and ICGC databases. The result of C-index (0.812), AUC (1-year, 0.836; 3-year, 0.767 and 5-year, 0.792) showed this nomogram predicted as well as ours (23). In other prognostic models including clinical and social characteristics for predicting mortality and/or recurrence for female breast cancer, they performed well in internal validation cohorts, but the results were unpredictable in external validation cohorts, especially in young and elderly patients, and in high risk patients (44). In our study, we have conducted various detailed analyses around GRPGs. For example, we provided the clinicopathological and survival information of the low-risk and high-risk groups and assessed the relationship between the expression level of each GRPG and OS in patients. Our study also showed GRPGs had a potential role in predicting response to immunotherapy in BC patients and we also displayed specific genetic alterations and differential analyses of the expression of 10 GRPGs in different molecular types of BC. These elaborate analyses were not available in the aforementioned studies.

GO functional and KEGG enrichment analyses were used to analyze the potential biological functions of the ten GRPGs. We found that the biological processes of GRPGs were mainly enriched in pyruvate metabolism, glycolysis, ATP generation from ADP, ADP metabolism, and nucleoside diphosphate phosphorylation. GRPGs in the signal pathways were enriched in carbon metabolism, biosynthesis of amino acids, and glycolysis/gluconeogenesis. Glycolysis is the foundation for carbon metabolism, which not only produces biomolecules for biosynthesis, but also provides ATP. GO and KEGG enrichment analyses showed that the GRPGs live up to their name. GSEA can integrate different data and can be used to evaluate the whole-genome expression profile of microarray data. GSVA is a nonparametric and unsupervised analysis method that can be used to evaluate gene set enrichment. In this study, GSEA and GSVA were conducted to analyze the enrichment of differentially expressed genes between the low-risk and high-risk groups. The results showed that several pathways were significantly enriched, indicating that GRPGs had a profound impact on BC biological functions. Furthermore, our study showed that GRPGs might be involved in regulating the TME and ICB response. TMB data and TIDE scores revealed that patients in the high-risk group were more likely to be sensitive to immunotherapy and benefit from ICB therapy. As immune and stromal cells play important roles in tumor growth, progression, and drug resistance, we used four scoring methods to estimate the abundance of immune and stromal cells. The stromal score, immune score, ESTIMATE score, and tumor purity showed different distributions between the high-risk and low-risk groups, indicating a higher abundance of cancer cells in the high-risk group than in the low-risk group. The ssGSEA algorithm was employed to estimate tumor infiltration, and up to 21 types of immune cell types were significantly lower in the high-risk group, which indicated that GRPGs had a significant effect on TME. Therefore, the GRPG-based prediction model is reliable for predicting the prognosis and immunotherapy efficacy, which may have potential implications in BC clinical practice.

Of the 10 GRPGs, ADP-dependent glucokinase (ADPGK) catalyzes ADP-dependent phosphorylation of glucose to glucose-6-phosphate and may play a role in glycolysis. Mutations in ADPGK have been shown to enhance BC cell migration and prompt metastasis *in vitro* experiments (45). Endoplasmic reticulum (ER)-localized ADPGK plays a critical role in T cell receptor (TCR)-induced the metabolic shift to aerobic glycolysis similar to the Warburg effect which is a common phenotype of activated immune cells (46). Heterogeneous nuclear ribonucleoprotein A1 (HNRNPA1) belongs to the A/B subfamily of ubiquitously expressed heterogeneous nuclear ribonucleoproteins (hnRNPs). This protein, along with other hnRNP proteins, is exported from the nucleus, probably bound to mRNA, and immediately re-imported. An isoform switch between the 3’-UTR isoforms of HNRNPA1 in BC has been found, and high HNRNPA1 protein levels correlate with poor survival in BC patients (47). HNRNPA1 is correlated with immunosuppressive status of the tumor immune microenvironment. Targeting HNRNPA1 can result in aberrant alternative splicing events and generation of immunogenic neoantigens that elicit anti-tumor immunity (48). Phosphoglycerate mutase 1 (PGAM1) is widely distributed in mammalian tissues and catalyzes the reversible conversion of 3-phosphoglycerate (3-PGA) to 2-phosphoglycerate (2-PGA) in the glycolytic pathway. PGAM1 expression is upregulated and related to poor prognosis in patients with BC (49). PGAM1 expression is positively correlated with infiltration levels of tumor-promoting immune cells such as macrophages, NK cells, and myeloid dendritic cells (50). In triple-negative breast cancer, PGAM1 is identified as a novel target that exhibits an antitumor effect via the regulation of immunocyte infiltration. PGAM1 inhibition synergizes with anti-PD-1 immunotherapy significantly remodeling the tumor microenvironment and leading to an increase in antitumor immunocytes and a reduction in immunosuppressive cell infiltration (51). The proviral integration site of Moloney murine leukemia virus 2 (PIM2) can promote glycolysis, BC tumorigenesis, and paclitaxel resistance through multiple mechanisms (52, 53). PIM2 plays a key role in immunomodulation, controls IL-15-mediated survival of natural killer cells and regulates early human Th17 cell differentiation (54, 55). In addition, proinflammatory macrophages trigger PIM2 expression in hepatocellular carcinoma cells which acquire the capability to survive, metastasize, and resist T-cell cytotoxicity and immunotherapy (56). Tyrosine 3-monooxygenase/tryptophan 5-monooxygenase activation protein zeta (YWHAZ) contributes to migration, chemotherapy resistance, and recurrence of BC (57, 58). Protein tyrosine kinase 2 (PTK2) is highly expressed in many cancers and is involved in cell growth, survival, migration, and invasion. A previous study confirmed that PTK2 can be used as a prognostic biomarker for BC and high PTK2 expression was correlated with infiltrating levels of multiple immune cells (59). Voltage-dependent anion channel 1 (VDAC1) is a major component of the outer mitochondrial membrane. It can be used as a cancer therapeutic target or diagnostic biomarker (60). VDAC1 mediates the release of mtDNA into the cytoplasm to enhance cytokine levels by activating immune responses and regulates mitochondrial Ca^2+^ transportation, lipid metabolism and mitophagy, which are involved in inflammation-related disease pathogenesis (61). Citrate synthase (CS) is a Krebs tricarboxylic acid cycle enzyme that catalyzes citrate synthesis from oxaloacetate and acetyl coenzyme A. CS inactivation facilitates aerobic glycolysis and cancer progression and targeting citrate can be regarded as a novel therapeutic strategy in cancer treatment (62, 63). Phosphoglycerate kinase 1 (PGK1) is a glycolytic enzyme that catalyses the conversion of 1,3-diphosphoglycerate to 3-phosphoglycerate. Higher PGK1 expression is associated with poor prognosis (64). Th17-cells of Crohn’s disease patients display heightened PGK1 and ALDOA and defective response to unconjugated bilirubin (65). In addition, PGK1 can be regarded as an immune target in Kawasaki disease (66). Glyceraldehyde-3-phosphate dehydrogenase spermatogenic (GAPDHS) plays a crucial role in carbohydrate metabolism and a novel GAPDH inhibitor can suppress BC growth effectively (67). GAPDH controls effector cytokine production by engaging/disengaging glycolysis and through fluctuations in its expression (68). A GAPDH serotonylation system has been reported recently to promote the glycolytic metabolism and antitumor immune activity of CD8+ T cells (69). The concordance of GAPDH expression in tumors with the TICs and immune checkpoints implies a certain association between GAPDH and the TME as well as cancer development (70). Collectively, these 10 GRPGs have been reported to participate in BC development and carcinogenesis (**Supplementary Table S9**).

The prognostic model we constructed has certain potential in patient stratification for immunotherapy and guiding treatment decisions. By evaluating risk scores, clinical doctors can better identify high-risk patients and provide valuable information in treatment choices, especially when developing personalized immunotherapy plans. However, this study had several limitations. First, although the TCGA-BRCA dataset provides rich information for large-scale studies, sample heterogeneity may affect the generalizability of the results. Second, despite using various statistical methods and bioinformatics tools to analyze the data, some potential biological signals may not have been fully captured due to limitations in sample size and grouping criteria. The complexity of the TME may affect our interpretation of indicators such as TMB and TIDE by multiple factors. In addition, in practical clinical applications, mRNA gene expression profiling analysis faces many challenges, such as variability and cost issues in sample collection and processing.

## 5 Conclusion

We identified 10 GRPGs and constructed an innovative and reliable prognostic model to predict OS and immunotherapeutic response in patients with BC. Moreover, a nomogram integrating this prediction model with clinical characteristics was created to predict the survival outcomes of patients with BC. Our study offers clinicians a bioinformatics tool to make individualized treatment plans and clinical decisions for patients with BC.

## Data Availability

The datasets (GEO data and TCGA-BRCA data) presented in the study are deposited in the GEO (https://www.ncbi.nlm.nih.gov/geo/, accession numbers: GSE20685, GSE42568, GSE29044) and TCGA (https://www.cancer.gov/about-nci/organization/ccg/research/structural-genomics/tcga, accession numbers: TCGA-BRCA).
