# Supplementary material for: A novel glycolysis-related gene signature for predicting prognosis and immunotherapy efficacy in breast cancer
Source: Front Immunol. 2025 Feb 19;16:1512859. doi: 10.3389/fimmu.2025.1512859 (PMC11880812; doi:10.3389/fimmu.2025.1512859)
Supplement: Supplementary file 1 [file Table1.docx]

**Table S1 The information of TCGA and GEO cohorts**

|  | **TCGA-BRCA** | **GSE20685** | **GSE42568** | **GSE29044** |
| --- | --- | --- | --- | --- |
| Platform | / | GPL570 | GPL570 | GPL570 |
| Species | Homo sapiens | Homo sapiens | Homo sapiens | Homo sapiens |
| Tissue | breast | breast | breast | breast |
| Samples in Tumor group | 1109 | 327 | 104 | 73 |
| Samples in Normal group | 113 | / | 17 | 36 |
| Reference |  | Correlation of microarray-based breast cancer molecular subtypes and clinical outcomes: implications for treatment optimization. | Correlating transcriptional networks to breast cancer survival: a large-scale co-expression analysis. | Age-specific gene expression signatures for breast tumors and cross-species conserved potential cancer progression markers in young women. |

GEO：Gene Expression Omnibus; BRCA：Breast Invasive Carcinoma; TCGA：The Cancer Genome Atlas

**Table S3 The Cox analyses of 10 GRPGs**

| Factor | Univariate analysis | |  | Multivariate analysis | |
| --- | --- | --- | --- | --- | --- |
|  | Hazard ratio (95% CI) | *P* value |  | Hazard ratio (95% CI) | *P* value |
| CS | 1.6 [1.11 - 2.29] | 0.0115 |  | 1.48 [0.994 - 2.2] | 0.0533 |
| PIM2 | 0.788 [0.656 - 0.945] | 0.0102 |  | 0.822 [0.681 - 0.993] | 0.0416 |
| PGK1 | 1.81 [1.44 - 2.28] | 4.83E-07 |  | 1.73 [1.29 - 2.31] | 0.000254 |
| GAPDHS | 3.93 [1.25 - 12.4] | 0.0195 |  | 4.16 [1.13 - 15.2] | 0.0315 |
| HNRNPA1 | 0.554 [0.383 - 0.8] | 0.00163 |  | 0.679 [0.46 - 1] | 0.0516 |
| ADPGK | 0.603 [0.391 - 0.93] | 0.0222 |  | 0.675 [0.437 - 1.04] | 0.0768 |
| YWHAZ | 1.28 [1.03 - 1.59] | 0.0254 |  | 0.865 [0.63 - 1.19] | 0.37 |
| PTK2 | 1.43 [1.1 - 1.86] | 0.0072 |  | 1.2 [0.849 - 1.71] | 0.298 |
| PGAM1 | 1.58 [1.12 - 2.22] | 0.00914 |  | 0.811 [0.524 - 1.26] | 0.349 |
| VDAC1 | 1.64 [1.19 - 2.27] | 0.00275 |  | 1.33 [0.922 - 1.92] | 0.127 |

**Table S4 GO enrichment analysis results**

| Ontology | ID | Description | GeneRatio | BgRatio | p.adjust | qvalue |
| --- | --- | --- | --- | --- | --- | --- |
| BP | GO:0006090 | pyruvate metabolic process | 5/10 | 106/18800 | 4.48E-07 | 2.71E-07 |
| BP | GO:0006096 | glycolytic process | 4/10 | 81/18800 | 7.63E-06 | 4.62E-06 |
| BP | GO:0006757 | ATP generation from ADP | 4/10 | 82/18800 | 7.63E-06 | 4.62E-06 |
| BP | GO:0046031 | ADP metabolic process | 4/10 | 90/18800 | 7.63E-06 | 4.62E-06 |
| BP | GO:0006165 | nucleoside diphosphate phosphorylation | 4/10 | 99/18800 | 7.63E-06 | 4.62E-06 |

GO: Gene Ontology; BP: Biological Process

**Table S5 KEGG enrichment analysis results**

| Ontology | ID | Description | GeneRatio | BgRatio | p.adjust | qvalue |
| --- | --- | --- | --- | --- | --- | --- |
| KEGG | hsa00010 | Glycolysis / Gluconeogenesis | 4/10 | 67/8164 | 4.86E-05 | 4.32E-05 |
| KEGG | hsa01200 | Carbon metabolism | 4/10 | 115/8164 | 0.000213 | 0.00019 |
| KEGG | hsa01230 | Biosynthesis of amino acids | 3/10 | 75/8164 | 0.00165 | 0.001467 |

KEGG: Kyoto Encyclopedia of Genes and Genomes

**Table S6 Patient Characteristics of BRCA patients in the BRCA datasets**

| Characteristics | Low | High | P value |
| --- | --- | --- | --- |
| n | 554 | 555 |  |
| T stage, n (%) |  |  | < 0.001 |
| T2 | 316 (28.6%) | 329 (29.8%) |  |
| T4 | 7 (0.6%) | 33 (3%) |  |
| T3 | 78 (7.1%) | 61 (5.5%) |  |
| T1 | 151 (13.7%) | 130 (11.8%) |  |
| N stage, n (%) |  |  | 0.371 |
| N1 | 191 (17.6%) | 178 (16.4%) |  |
| N3 | 34 (3.1%) | 44 (4%) |  |
| N2 | 55 (5.1%) | 65 (6%) |  |
| N0 | 270 (24.8%) | 251 (23.1%) |  |
| M stage, n (%) |  |  | 0.045 |
| M0 | 454 (41%) | 464 (42%) |  |
| M1 | 6 (0.5%) | 16 (1.4%) |  |
| MX | 91 (8.2%) | 75 (6.8%) |  |
| Pathologic stage, n (%) |  |  | 0.016 |
| Stage II | 321 (29.3%) | 311 (28.4%) |  |
| Stage III | 116 (10.6%) | 135 (12.3%) |  |
| Stage I | 102 (9.3%) | 80 (7.3%) |  |
| Stage IV | 5 (0.5%) | 15 (1.4%) |  |
| Stage X | 3 (0.3%) | 9 (0.8%) |  |
| Age, n (%) |  |  | < 0.001 |
| <=60 | 345 (31.1%) | 269 (24.3%) |  |
| >60 | 209 (18.8%) | 286 (25.8%) |  |
| OS, n (%) |  |  | < 0.001 |
| No | 499 (45%) | 453 (40.9%) |  |
| Yes | 54 (4.9%) | 102 (9.2%) |  |
| DSS, n (%) |  |  | < 0.001 |
| No* | 518 (47.6%) | 483 (44.4%) |  |
| Yes* | 28 (2.6%) | 59 (5.4%) |  |
| PFI, n (%) |  |  | 0.004 |
| No | 495 (44.7%) | 464 (41.9%) |  |
| Yes | 58 (5.2%) | 91 (8.2%) |  |
| ER, n (%) |  |  | 0.612 |
| ER- | 117 (11.1%) | 123 (11.6%) |  |
| ER+ | 414 (39.1%) | 404 (38.2%) |  |
| PR, n (%) |  |  | 0.137 |
| PR- | 162 (15.4%) | 185 (17.5%) |  |
| PR+ | 365 (34.6%) | 343 (32.5%) |  |
| HER2, n (%) |  |  | < 0.001 |
| HER2- | 305 (41.6%) | 264 (36%) |  |
| HER2+ | 54 (7.4%) | 110 (15%) |  |
| TNBC, n (%) |  |  | 0.114 |
| TNBC | 66 (6%) | 50 (4.5%) |  |
| Non-TNBC | 488 (44%) | 505 (45.5%) |  |

*No: Alive; Yes: Dead

**Table S7 GSVA enrichment analysis results of TCGA-BRCA**

| Description | logFC | p.adjust |
| --- | --- | --- |
| WP_AEROBIC_GLYCOLYSIS | -0.40514 | 2.70E-42 |
| REACTOME_FOLDING_OF_ACTIN_BY_CCT_TRIC | -0.39428 | 3.70E-32 |
| FU_INTERACT_WITH_ALKBH8 | -0.37246 | 6.56E-33 |
| WP_GLYCOLYSIS_IN_SENESCENCE | -0.37056 | 8.01E-40 |
| IKEDA_MIR1_TARGETS_DN | -0.36731 | 1.34E-36 |
| REACTOME_GOLGI_CISTERNAE_PERICENTRIOLAR_STACK_REORGANIZATION | -0.3669 | 1.31E-39 |
| SMID_BREAST_CANCER_NORMAL_LIKE_DN | -0.36628 | 4.76E-26 |
| REACTOME_UPTAKE_AND_FUNCTION_OF_DIPHTHERIA_TOXIN | -0.36231 | 6.17E-33 |
| FARMER_BREAST_CANCER_CLUSTER_2 | -0.35879 | 7.24E-24 |
| REACTOME_DISEASES_OF_MISMATCH_REPAIR_MMR | -0.34904 | 6.18E-22 |
| NAKAMURA_ALVEOLAR_EPITHELIUM | 0.30714 | 1.08E-19 |
| REACTOME_INTERLEUKIN_18_SIGNALING | 0.307417 | 2.64E-24 |
| REACTOME_MITOCHONDRIAL_UNCOUPLING | 0.308522 | 1.74E-27 |
| REACTOME_GLI_PROTEINS_BIND_PROMOTERS_OF_HH_RESPONSIVE_GENES_TO_PROMOTE_TRANSCRIPTION | 0.313096 | 1.04E-20 |
| REACTOME_EICOSANOID_LIGAND_BINDING_RECEPTORS | 0.318627 | 2.12E-36 |
| LOPEZ_MESOTHELIOMA_SURVIVAL_OVERALL_UP | 0.329856 | 6.81E-27 |
| CHASSOT_SKIN_WOUND | 0.33721 | 2.40E-20 |
| REACTOME_FLT3_SIGNALING_THROUGH_SRC_FAMILY_KINASES | 0.343559 | 3.59E-23 |
| MILICIC_FAMILIAL_ADENOMATOUS_POLYPOSIS_DN | 0.350475 | 1.02E-28 |
| GARGALOVIC_RESPONSE_TO_OXIDIZED_PHOSPHOLIPIDS_LIGHTGREEN_DN | 0.41217 | 3.13E-48 |

GSVA: Gene Set Variation Analysis

**Table S8 GSEA enrichment analysis results of TCGA-BRCA**

| Description | setSize | NES | p.adjust | qvalues |
| --- | --- | --- | --- | --- |
| KEGG_PRIMARY_IMMUNODEFICIENCY | 35 | 1.719596 | 0.00829 | 0.00758 |
| WP_FOLATE_METABOLISM | 66 | 1.508646 | 0.046376 | 0.042403 |
| REACTOME_CELLULAR_SENESCENCE | 193 | -2.25764 | 3.97E-09 | 3.63E-09 |
| REACTOME_OXIDATIVE_STRESS_INDUCED_SENESCENCE | 121 | -2.53145 | 3.97E-09 | 3.63E-09 |
| REACTOME_DNA_METHYLATION | 62 | -2.57911 | 3.97E-09 | 3.63E-09 |
| REACTOME_RMTS_METHYLATE_HISTONE_ARGININES | 76 | -2.58742 | 3.97E-09 | 3.63E-09 |
| REACTOME_HDACS_DEACETYLATE_HISTONES | 91 | -2.59168 | 3.97E-09 | 3.63E-09 |
| REACTOME_CONDENSATION_OF_PROPHASE_CHROMOSOMES | 71 | -2.59468 | 3.97E-09 | 3.63E-09 |
| REACTOME_ERCC6_CSB_AND_EHMT2_G9A_POSITIVELY_REGULATE_RRNA_EXPRESSION | 73 | -2.60659 | 3.97E-09 | 3.63E-09 |
| REACTOME_BINDING_AND_UPTAKE_OF_LIGANDS_BY_SCAVENGER_RECEPTORS | 98 | 2.302314 | 3.97E-09 | 3.63E-09 |
| REACTOME_SCAVENGING_OF_HEME_FROM_PLASMA | 69 | 2.279959 | 3.97E-09 | 3.63E-09 |
| REACTOME_CD22_MEDIATED_BCR_REGULATION | 61 | 2.265495 | 3.97E-09 | 3.63E-09 |
| REACTOME_ANTIGEN_ACTIVATES_B_CELL_RECEPTOR_BCR_LEADING_TO_GENERATION_OF_SECOND_MESSENGERS | 86 | 2.265251 | 3.97E-09 | 3.63E-09 |
| REACTOME_FCERI_MEDIATED_NF_KB_ACTIVATION | 136 | 2.236623 | 3.97E-09 | 3.63E-09 |
| REACTOME_FCERI_MEDIATED_MAPK_ACTIVATION | 87 | 2.234211 | 3.97E-09 | 3.63E-09 |

GSEA: Gene Set Enrichment Analysis

**Table S9 The functions of 10 GRPGs in** **breast cancer in previous reports**

| Gene | Biological function and clinical significance in BC | molecular subtype | References |
| --- | --- | --- | --- |
| CS | CS inactivation facilitates glycolysis and cancer progression, targeting citrate is a novel strategy | Luminal/TNBC | 62 |
| PIM2 | PIM2 interacts with tristetraprolin and promotes BC tumorigenesis; PIM2 regulates PFKFB3 to promote glycolysis and paclitaxel resistance | Luminal/TNBC | 52;53 |
| PGK1 | PGK1 expression is associated with poor prognosis and pro-tumor immunity | Luminal/TNBC/HER2+ | 64 |
| GAPDHS | DC-5163 is a promising GAPDH inhibitor for suppressing BC growth | Luminal/TNBC | 67 |
| HNRNPA1 | An mRNA isoform switch is identified and high protein levels correlate with poor survival | Luminal/HER2+/TNBC | 47 |
| ADPGK | Mutations in ADPGK can enhance cell migration and prompt metastasis | Luminal/HER2+/TNBC | 45 |
| YWHAZ | YWHAZ contributes to migration, chemotherapy resistance, and recurrence of BC | Luminal/TNBC/HER2+ | 57;58 |
| PTK2 | PTK2 is an adverse prognostic biomarker | Luminal/HER2+/TNBC | 59 |
| PGAM1 | PGAM1 negatively regulates ASS1 expression; PGAM1 inhibition synergizes with anti-PD-1 immunotherapy significantly remodeling the tumor microenvironment | Luminal/TNBC; TNBC | 49;51 |
| VDAC1 | VDAC1 can be used as a therapeutic target or diagnostic biomarker | Luminal/TNBC/HER2+ | 60 |
